# EspL is essential for virulence and stabilizes EspE, EspF and EspH levels in *Mycobacterium tuberculosis*

**DOI:** 10.1371/journal.ppat.1007491

**Published:** 2018-12-20

**Authors:** Claudia Sala, Nina T. Odermatt, Paloma Soler-Arnedo, Muhammet F. Gülen, Sofia von Schultz, Andrej Benjak, Stewart T. Cole

**Affiliations:** Global Health Institute, Ecole Polytechnique Fédérale de Lausanne, Lausanne, Switzerland; University of Massachusetts Medical School, UNITED STATES

## Abstract

The ESX-1, type VII, secretion system represents the major virulence determinant of *Mycobacterium tuberculosis*, one of the most successful intracellular pathogens. Here, by combining genetic and high-throughput approaches, we show that EspL, a protein of 115 amino acids, is essential for mediating ESX-1-dependent virulence and for stabilization of EspE, EspF and EspH protein levels. Indeed, an *espL* knock-out mutant was unable to replicate intracellularly, secrete ESX-1 substrates or stimulate innate cytokine production. Moreover, proteomic studies detected greatly reduced amounts of EspE, EspF and EspH in the *espL* mutant as compared to the wild type strain, suggesting a role for EspL as a chaperone. The latter conclusion was further supported by discovering that EspL interacts with EspD, which was previously demonstrated to stabilize the ESX-1 substrates and effector proteins, EspA and EspC. Loss of EspL also leads to downregulation in *M*. *tuberculosis* of WhiB6, a redox-sensitive transcriptional activator of ESX-1 genes. Overall, our data highlight the importance of a so-far overlooked, though conserved, component of the ESX-1 secretion system and begin to delineate the role played by EspE, EspF and EspH in virulence and host-pathogen interaction.

## Author summary

*Mycobacterium tuberculosis* is the etiological agent of human tuberculosis, a life-threatening disease which has seen a recrudescence in the last decades due to the spread of drug-resistant bacterial strains and to co-morbidities such as HIV and diabetes. To develop effective treatment and limit bacterial dissemination within and outside the host, it is pivotal to improve our understanding of the strategies used by the pathogen to colonize the host and subvert the immune defenses. The ESX-1 secretion system represents a key player in these processes. Here we show that the EspL protein, encoded by the ESX-1 gene cluster, is essential for bacterial virulence and for stabilizing the abundance of the EspE, EspF and EspH components of the ESX-1 system. Tubercle bacilli lacking EspL cannot multiply inside macrophages, do not secrete the major virulence factor EsxA and fail to trigger the ESX-1 dependent innate immune response. EspL is thus an important but so-far neglected contributor to ESX-1 function.

## Introduction

*Mycobacterium tuberculosis*, the etiological agent of human tuberculosis, is arguably the world’s most successful human pathogen. It is estimated that one third of the world’s population is latently infected by the bacterium [1], which can survive in a dormant state inside specialized cellular structures in the lung parenchyma called granulomas [2,3]. As a consequence of immunodeficiency or co-morbidities, like HIV or diabetes [4,5], latent *M*. *tuberculosis* can reactivate and establish an acute infectious process which leads to the disease. Host-pathogen interaction and disease progression are mediated by various virulence factors encoded by the bacterial genome, the most important of them being the ESX-1 or type VII secretion system [6].

ESX loci are characterized by genes encoding small secreted proteins with a conserved tryptophan-x-glycine (WXG) motif and by transmembrane ATPases belonging to the FtsK-SpoIIIE-like family [7,8]. Five ESX systems, implicated in different functions, exist in *M*. *tuberculosis* [9]. The ESX-1 cluster comprises approximately twenty genes and encodes a specialized secretion apparatus, which releases effectors into the extracellular milieu. The relevance of the ESX-1 genes in mycobacterial physiology was recognized when attenuation of the vaccine strain *M*. *bovis* BCG and of the vole bacillus *M*. *microti* was associated with their partial deletion [10–14]. Since then, the role played by ESX-1 in cytosolic recognition and stimulation of innate immunity [15–17], phagosomal rupture and bacterial escape [18,19], intercellular spread and systemic disease [20,21] has been the object of numerous studies. Recently, the ESX-1 secretion system has also been considered as a potential drug target for the development of anti-virulence drugs [22].

Considerable progress has been made in understanding how the system works and is regulated. Electron microscopy-based studies showed that the *M*. *xenopi* ESX-5 core membrane complex is composed of four proteins (EccB_5_, the ATPase EccC_5_, the putative channel EccD_5_, and EccE_5_) which assemble into an oligomer with a six-fold symmetry [23]. However, it is still unknown how the secreted substrates can cross the mycobacterial external membrane, or mycomembrane, although the involvement of EspC, encoded by the distal *espA-espC-espD* locus, has been hypothesized in *M*. *tuberculosis* [24]. The current model for ESX-1 activity proposes heterodimeric and co-dependent complexes as the secreted substrates, i.e. EsxA/EsxB and EspA/EspC [25,26]. These are targeted to the inner membrane apparatus by a bipartite secretory signal composed of the WXG motif on the first member of the dimer and of a tyrosine-x-x-x-aspartic acid/glutamic acid (YxxxD/E) motif on the second [26,27]. ESX-1 function undergoes transcriptional regulation, exerted by EspR [28], Lsr2 [29], CRP [30], MprA [31] and mIHF [32] on the *espA-espC-espD* locus. Recent studies showed that WhiB6, a redox sensor protein, directly controls expression of genes associated with the ESX-1 secretion system in *M*. *marinum*, such as *espA*, *espE* and *eccA1*, and that this regulation is strictly dependent on its Fe-S cluster [33]. In *M*. *tuberculosis*, *whiB6* is part of the PhoP regulon [34] and divergently contributes to ESX-1 gene transcription in the H37R strains compared to other isolates [35]. Additional, post-transcriptional, control of secretion activity is carried out by the serine protease MycP1 through proteolytic cleavage of another ESX-1 substrate, EspB [36].

Here, we investigate the role of a previously overlooked ESX-1 component, EspL, in *M*. *tuberculosis*. We demonstrate that it is essential for mycobacterial replication inside macrophages, for eliciting innate cytokine production and for stabilizing the protein levels of the additional ESX-1 members EspE, EspF and EspH.

## Results

### Construction of an *espL* deletion mutant

**Fig 1:**
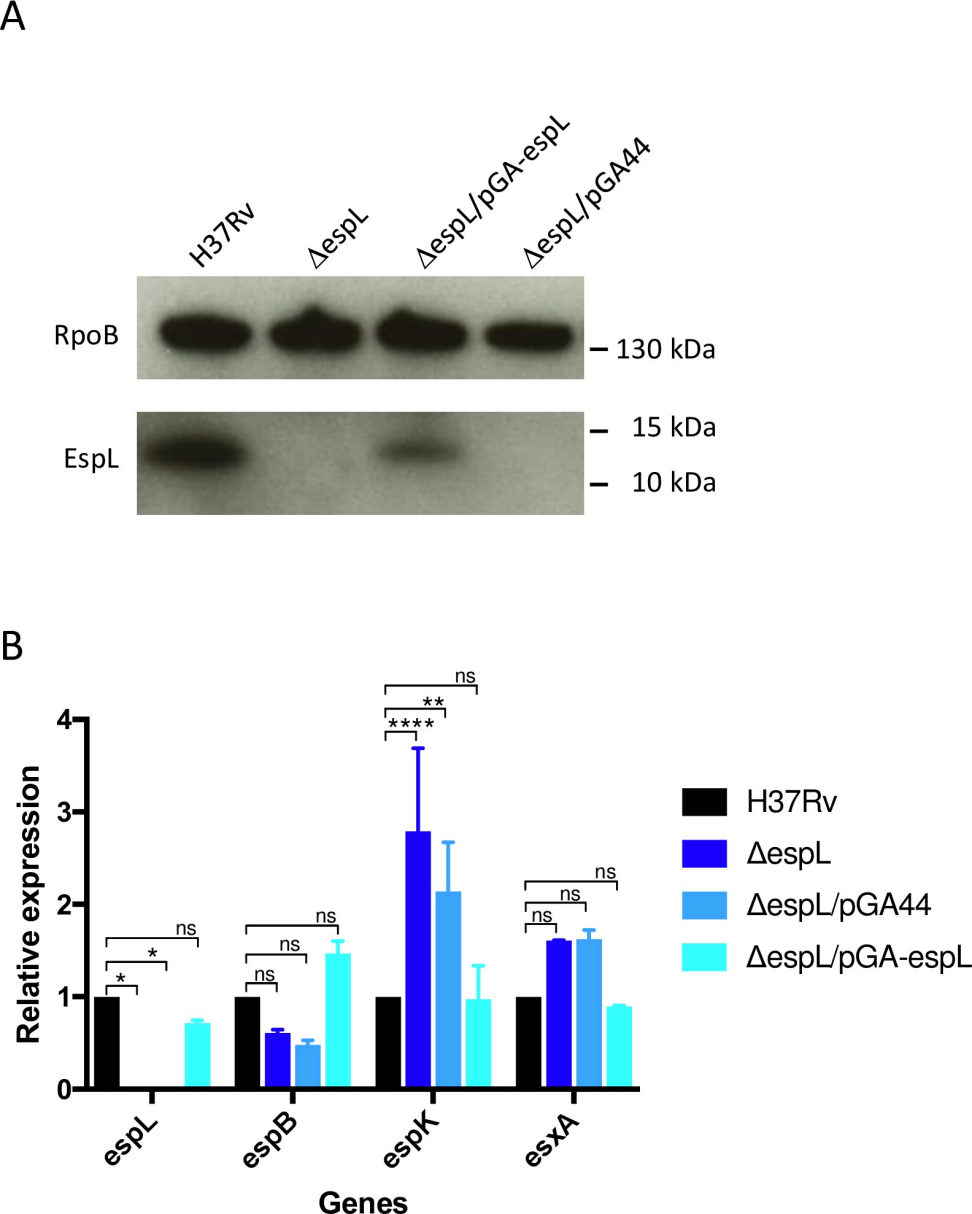
Validation of Δ*espL* mutant. **A)** Detection of EspL by immunoblot analysis of total protein extracts from *M*. *tuberculosis* H37Rv, Δ*espL* mutant and complemented strain. RpoB was used as a loading control. **B)** qRT-PCR analysis of *espL*, *espB*, *espK* and *esxA* gene expression levels in different strains. Data were obtained from two independent replicates, normalized to the housekeeping gene *sigA* and expressed as relative to H37Rv. *, *p* < 0.05. **, *p* < 0.005. ****, *p* < 0.0001. ns, not significant in two-way ANOVA followed by Tukey’s multiple comparison test.

In order to evaluate the role of EspL in *M*. *tuberculosis* virulence and ESX-1-dependent secretion activity, construction of an unmarked deletion mutant was planned. The transcriptional profile of the H37Rv genomic region that includes *espL* was carefully considered to avoid polarity on the downstream gene *espK*. Studies by Cortes and colleagues [37] demonstrated the presence of a polycistronic RNA that covers *mycP1*, *eccE1*, *espB* and *espL*, but not *espK*, which is transcribed independently (S1A Fig). Additionally, sequence inspection revealed that the GTG translational start codon of *espL* overlaps the stop codon of the preceding gene *espB*. The pJG1100-derived suicide vector [38] was then constructed according to this pre-existing information and the *espL* coding sequence (CDS) was deleted from the chromosome by allelic exchange, from coordinate 4,360,199 to coordinate 4,360,543, thereby leaving the *espB* stop codon intact (S1B Fig). The resulting strain, named Δ*espL*, was validated by immunoblot (Fig 1A) and whole genome sequencing. The latter technology identified one single nucleotide polymorphism (SNP) in gene *rv1403* (353T>C), which caused substitution of Val118 with Ala, and one SNP in *ethA* (A to G transition at position 368) which resulted in replacement of His123 with Arg. No other differences were noted upon comparison with the genome sequence of the parental strain, except for the intended deletion. Δ*espL* was transformed with the complementing plasmid pGA44-*espL*, which carries the *espL* gene under the control of the PTR promoter, or with the empty vector pGA44 as a control [39]. Immunoblot experiments proved that expression of EspL was restored in the complemented strain Δ*espL*/pGA-*espL*, whereas no band was detected in the control Δ*espL*/pGA44 (Fig 1A). Consistent with these findings, qRT-PCR showed that the *espL* mRNA was expressed at a level similar to that of the wild type when the gene was provided *in trans* (Fig 1B). Transcriptional analysis was extended to include *espB*, *espK* and *esxA*. While *espB* and *esxA* mRNA levels were not altered significantly by the mutation introduced or by the ectopic expression of *espL*, the amount of *espK* transcript was 2.5-3-fold higher in the mutant strain (Fig 1B).

### Δ*espL* is attenuated and does not stimulate innate immunity *ex vivo*

**Fig 2:**
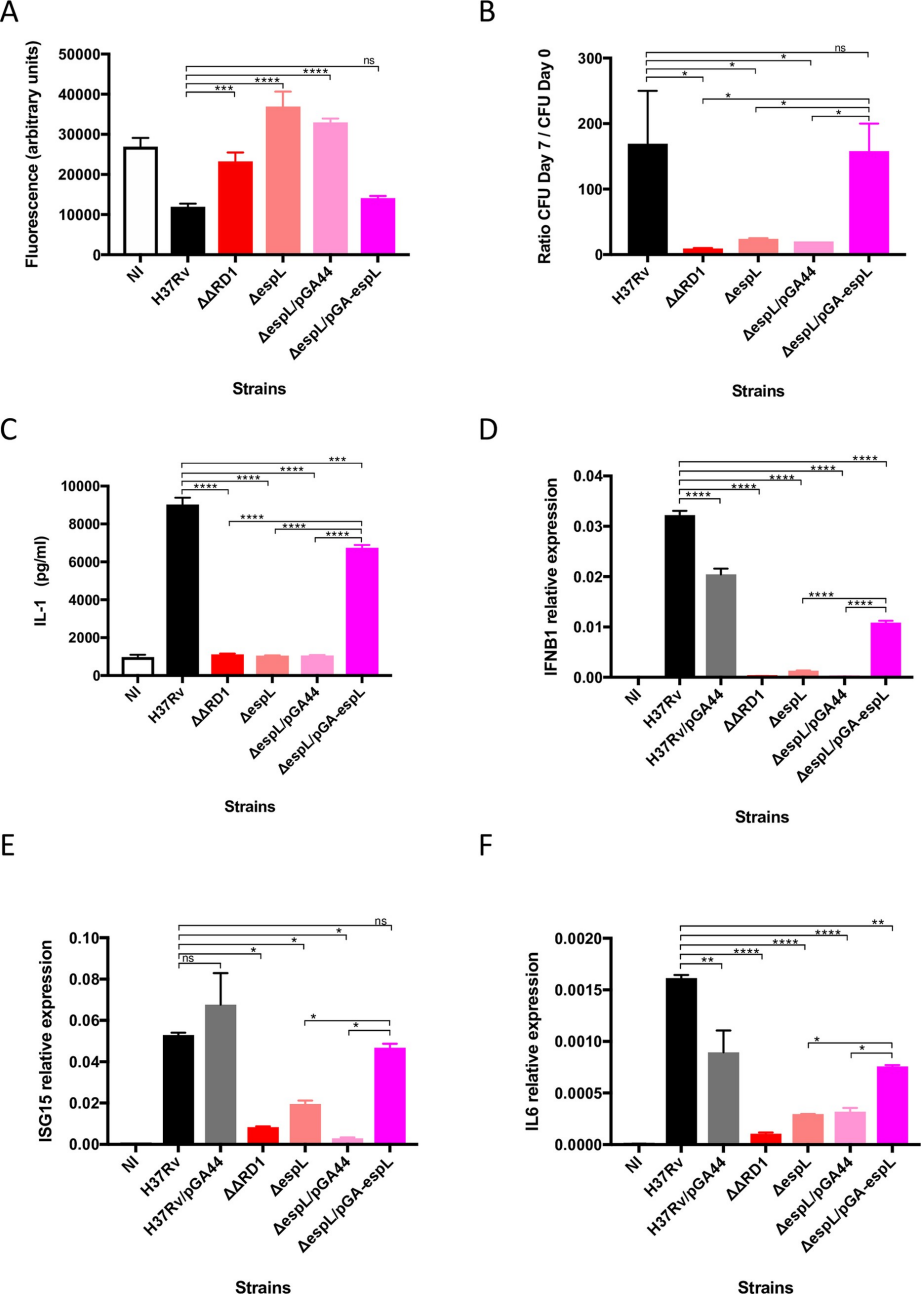
*Ex vivo* phenotype of Δ*espL* mutant. **A)** Virulence of Δ*espL* mutant compared to H37Rv and complemented strain in the THP-1 infection model. ΔΔRD1 carries a deletion of the extended ESX-1 locus. THP-1 cells were infected at multiplicity of infection (MOI) of 5. Fluorescence measurements directly correlate with THP-1 viability. Data were expressed as the mean and standard deviation (SD) of four independent replicates. **B)** Colony forming unit (CFU) evaluation of intracellular bacteria upon THP-1 infection. THP-1 cells were infected at multiplicity of infection (MOI) of 20:1 (cells:bacteria). Data were expressed as the mean and SD of two independent replicates. **C), D), E) and F)** Cytokine expression levels measured upon THP-1 infection with different bacterial strains. IL-1β was detected by ELISA assays. *IFNB*, *ISG15* and *IL6* were quantified by qRT-PCR. Data were expressed as the mean and SD of two independent replicates. NI: not infected control. *, *p* < 0.05. **, *p* < 0.005. ***, *p* < 0.0005. ****, *p* < 0.0001. ns, not significant in one-way ANOVA followed by Tukey’s multiple comparison test.

The Δ*espL* mutant did not show any major difference as compared to the wild type strain during *in vitro* growth in standard medium (S2A Fig). However, infection of THP-1 cells demonstrated severe reduction of the cytotoxicity of the mutant, which allowed cell survival to a similar extent as upon infection with the ΔΔRD1 strain, which lacks the extended RD1 region [40] (Fig 2A). Importantly, expression of *espL* by pGA44 complemented the phenotype to wild type levels (Fig 2A). These findings were further confirmed by colony forming unit (CFU) enumeration. While all of the strains were equally phagocytosed by THP-1 cells (S2B Fig), a major increase in the number of intracellular bacteria over one week was reported when H37Rv and the complemented strains, but not Δ*espL*, were used for infection (Fig 2B).

The crucial role played by the ESX-1 secretion system in inducing expression of cytokines of the innate immunity pathways was previously illustrated. In particular, EsxA secretion was found to be responsible for activating the cytosolic surveillance pathway based on cGAS-dependent sensing of DNA and the inflammasome [15–17]. Here, markedly reduced production of the pro-inflammatory cytokine IL-1β (Fig 2C), as well as decreased expression of type I interferon gene *IFNB1* (Fig 2D), interferon-stimulated gene *ISG15* (Fig 2E), and interleukin gene *IL6* (Fig 2F), were noted after THP-1 infection by the Δ*espL* strain. On the contrary, H37Rv and the complemented strain Δ*espL*/pGA-*espL* elicited production of cytokines belonging to both the cGAS-STING-type I Interferon (IFN) and inflammasome axes. Overall, these data indicate that EspL is a key player in *M*. *tuberculosis* virulence, interaction with the immune system and, likely, in ESX-1 secretion, as explained below.

### Secretion of ESX-1 substrates is compromised in Δ*espL*

**Fig 3:**
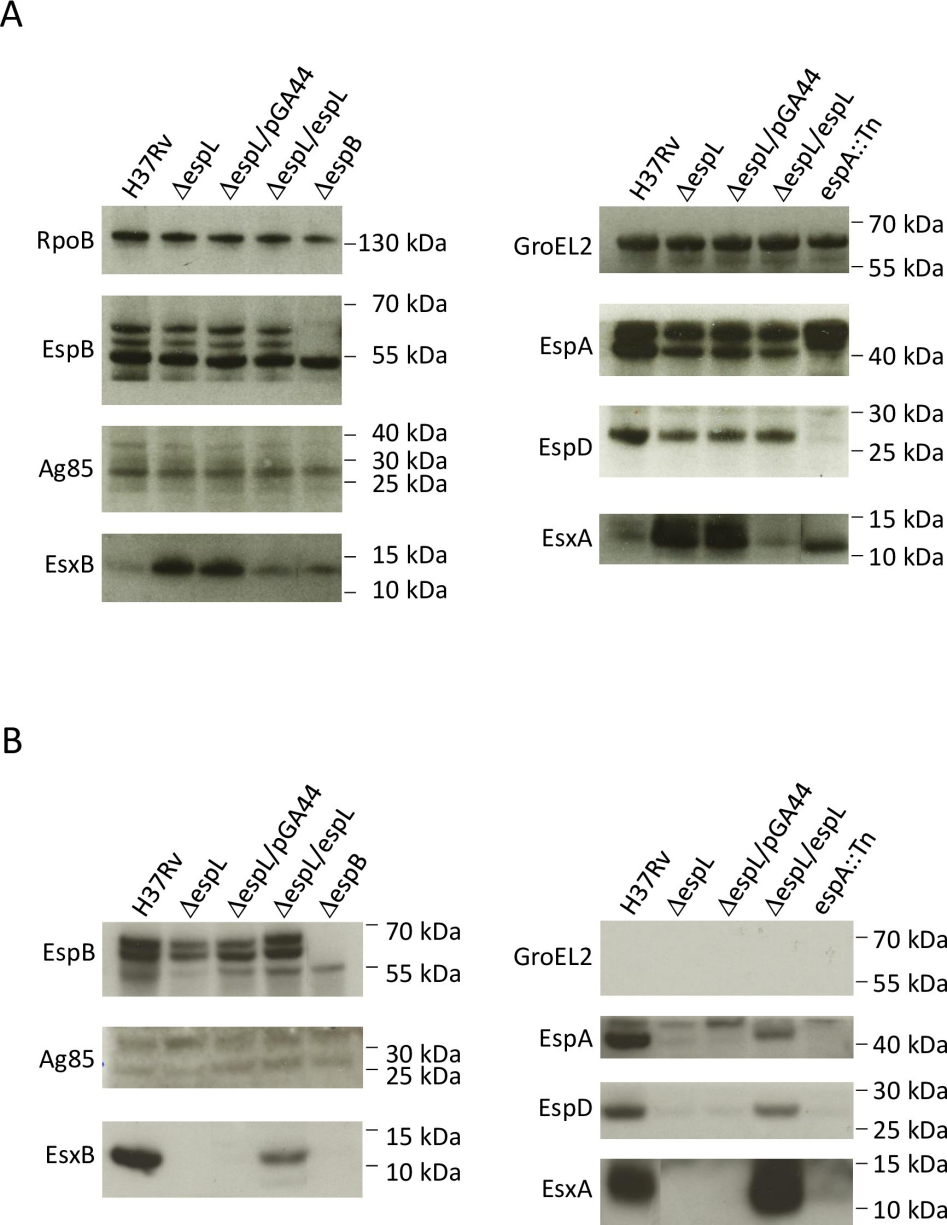
Immunoblot analysis of Δ*espL* mutant. **A)** Total cell lysates prepared from the indicated bacterial strains were analyzed by immunoblot. Membranes were probed for RpoB, which represented the loading control, EspB, Antigen 85 (Ag85), EsxB, GroEL2, EspA, EspD and EsxA. **B)** Culture filtrates were analyzed as described for the total cell lysates. The experiment was repeated two times. One representative image is shown.

The secretion profile of the mutant was examined in parallel to that of the wild type H37Rv and of the complemented mutant strains. While the proteins were produced by all of the strains (Fig 3A), EsxA and EsxB were not detectable in the secreted fraction by immunoblot when EspL was missing, whereas EspA and EspD levels were greatly reduced (Fig 3B). As a consequence of the compromised secretion, accumulation of EsxA and EsxB occurred inside Δ*espL* cells (Fig 3A). Interestingly, EspB was found to be released into the culture supernatant in the absence of EspL (Fig 3B and S3 Fig), confirming that its secretion is not dependent on EsxA, EsxB, EspA or EspD, as reported earlier [41]. Therefore, the severe attenuation of Δ*espL*, described above, correlates with lack of secretion of the major virulence factor EsxA.

In-depth analysis of the culture filtrates of strains H37Rv, Δ*espL*, Δ*espL*/pGA-*espL* was performed by mass-spectrometry (S4 Fig, S2 and S4 Tables). While confirming the findings described above, these additional experiments showed that EspE, EspF and EspH (and other ESX-1 substrates such as EspC) are underrepresented in the secreted fraction of the mutant strain, indicating that ESX-1-dependent secretion activity is affected in Δ*espL*.

### Localization of EspL in sub-cellular fractions

To gain insight into EspL function, the localization of the protein in sub-cellular fractions was studied. Total extracts from strain H37Rv were separated into cytosolic, membrane and capsular proteins, in addition to culture filtrate preparations. Anti-EspL antibodies identified a protein, with the apparent molecular weight of EspL, mainly in the cytosol and, to a lesser extent, in the membrane. However, partial contamination of the membrane fraction could not be excluded (S5 Fig). EspL was undetectable in the culture filtrate. Control antibodies against RpoB, Rv3852 and EsxB recognized their cognate antigens in the cytosolic/membrane, membrane only or cytosolic/secreted fractions, as expected [42]. EspL could thus exert its function in the cytosol or as a membrane-associated protein.

### Deletion of *espL* causes reduced expression of *whiB6*

Since PFAM [43] and recent experimental work [44] predicted the presence of an YbaB-type DNA-binding domain [45–47] in EspL, we hypothesized that the protein may influence gene expression through binding to DNA. The transcriptome of the mutant strain was then analyzed and compared to that of the wild type by RNA-seq. Despite the low cut-off value (2-fold change), none of the *M*. *tuberculosis* genes was found to be deregulated in Δ*espL*, except for *whiB6*, whose expression level was decreased by 3-fold on average (S1 Table), and *espL* itself, which was not detected in the knock-out mutant. Genes belonging to the ESX-1 cluster, as well as genes which are part of other ESX loci (ESX-2 to ESX-5) were expressed at similar levels in the mutant as compared to the wild type. Curiously, genes that were reported to be included in the WhiB6 regulon in the related species *Mycobacterium marinum* [33] were not found to be deregulated by RNA-seq in *M*. *tuberculosis* Δ*espL* (S1 Table). These findings were confirmed independently by qRT-PCR, which also proved that re-introduction of *espL* into the complemented strain was necessary and sufficient to restore *espL* and *whiB6* mRNA levels to normal (S6 Fig). Thus, EspL seems to control expression of *whiB6* either directly or indirectly.

### Ectopic expression of *whiB6* in Δ*espL* increases ESX-1 gene expression levels but does not complement attenuation

**Fig 4:**
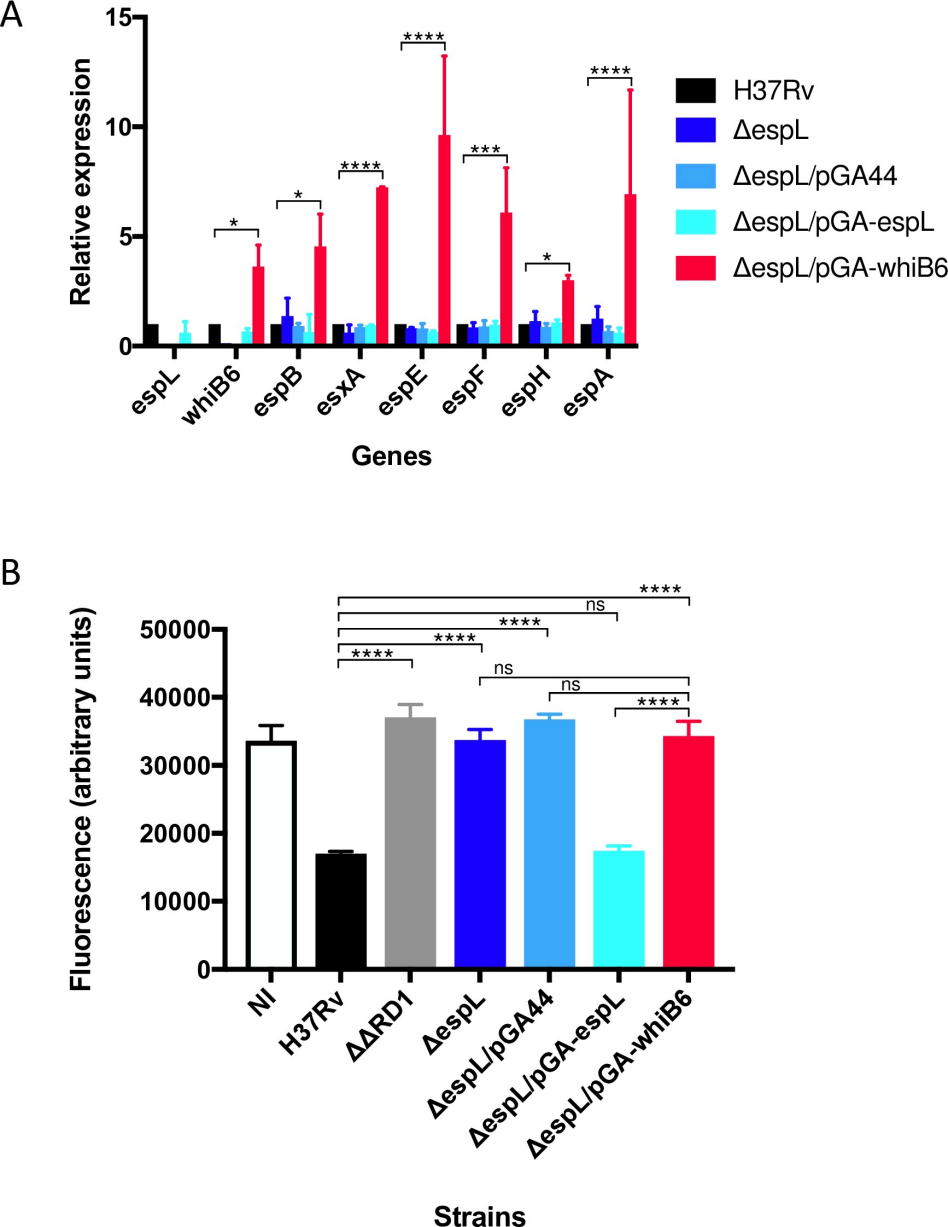
Phenotype obtained upon expression of *whiB6* in the Δ*espL* mutant. **A)** qRT-PCR analysis of the expression levels of the indicated genes in different strains. Data were obtained from two independent replicates, normalized to the housekeeping gene *sigA* and expressed as relative to H37Rv. *, *p* < 0.05. ***, *p* < 0.0005. ****, *p* < 0.0001 in two-way ANOVA followed by Tukey’s multiple comparison test. **B)** Virulence of Δ*espL* mutant expressing *whiB6 in trans* compared to H37Rv, Δ*espL* and complemented strain in the THP-1 infection model. ΔΔRD1 carries a deletion of the extended ESX-1 locus. THP-1 cells were infected at multiplicity of infection (MOI) of 5. Fluorescence measurements directly correlate with THP-1 viability. Data were expressed as the mean and standard deviation (SD) of four independent replicates. NI: not infected control. ****, *p* < 0.0001. ns, not significant in one-way ANOVA followed by Tukey’s multiple comparison test.

Intrigued by the discoveries reported earlier, we examined the impact of constitutive expression of *whiB6* in the Δ*espL* mutant. Levels of *whiB6* mRNA were increased by approximately 4-fold in the Δ*espL* strain carrying pGA-*whiB6*, as compared to wild type. All of the tested genes (i.e. *espB*, *esxA*, *espE*, *espF*, *espH* and *espA*), which are part of different transcriptional units [37,48], were induced (Fig 4A), therefore indicating that WhiB6 works as an activator of ESX-1 genes in *M*. *tuberculosis*. However, despite the increased expression of virulence-related genes, Δ*espL*/pGA-*whiB6* displayed the same attenuation as Δ*espL*, Δ*espL*/pGA44 and ΔΔRD1 (Fig 4B). In other words, the lack of cytotoxicity caused by deletion of *espL* could not be bypassed by ectopic over-expression of *whiB6*. EspL is therefore essential for *M*. *tuberculosis* virulence.

### EspL stabilizes the cytosolic levels of EspE, EspF and EspH

**Fig 5:**
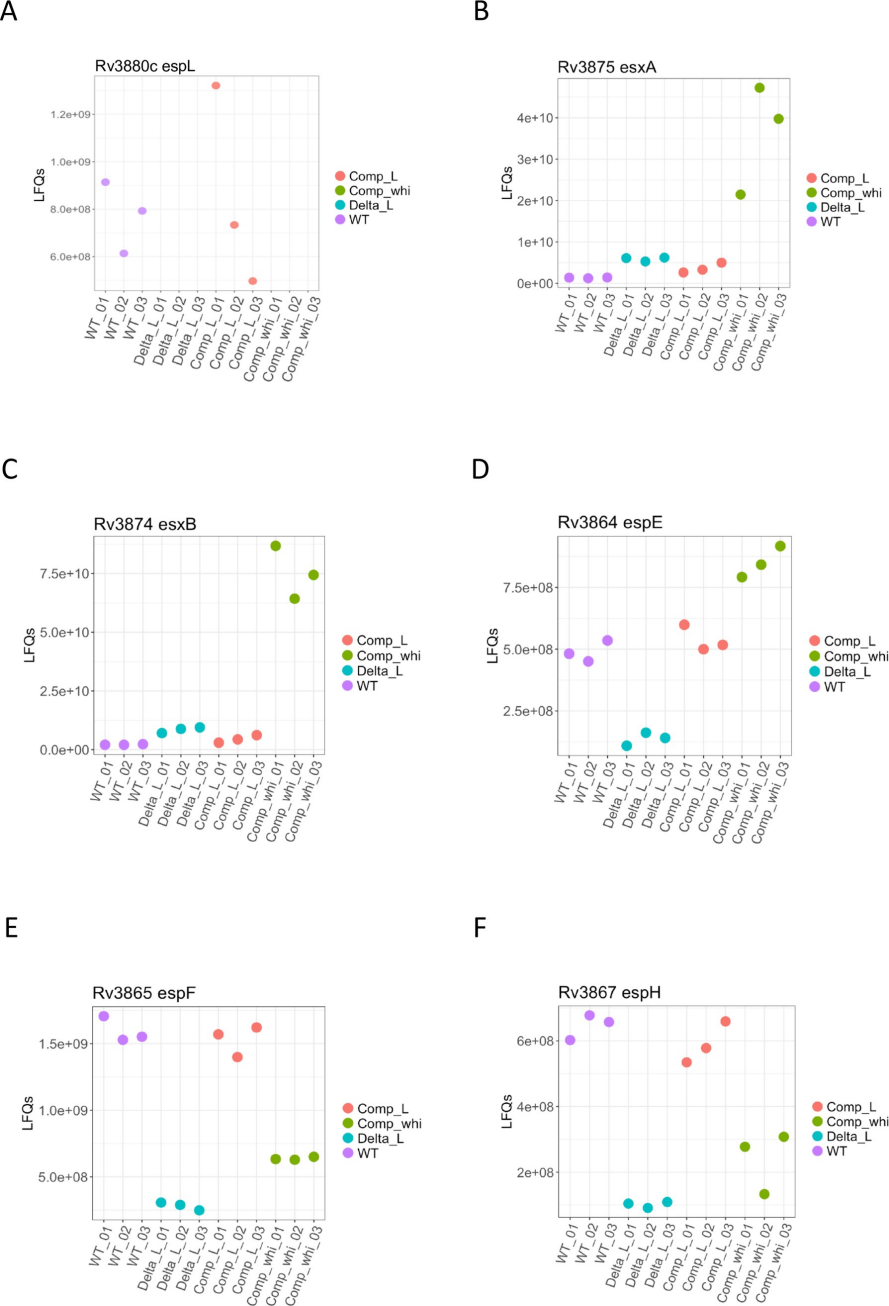
Mass spectrometry analysis of protein abundance in the Δ*espL* mutant. Total protein extracts were prepared in triplicate and subjected to Mass Spectrometry analysis. Each graph shows the abundance of the indicated protein in the three replicates in the various strains. **A)** EspL. **B)** EsxA. **C)** EsxB. **D)** EspE. **E)** EspF. **F)** EspH. WT: H37Rv. Delta_L: Δ*espL*. Comp_L: Δ*espL*/*espL* (complemented strain). Comp_whi: Δ*espL*/*whiB6* (strain expressing *whiB6 in trans*). Data are expressed as LFQs (Label Free Quantifications).

**Fig 6:**
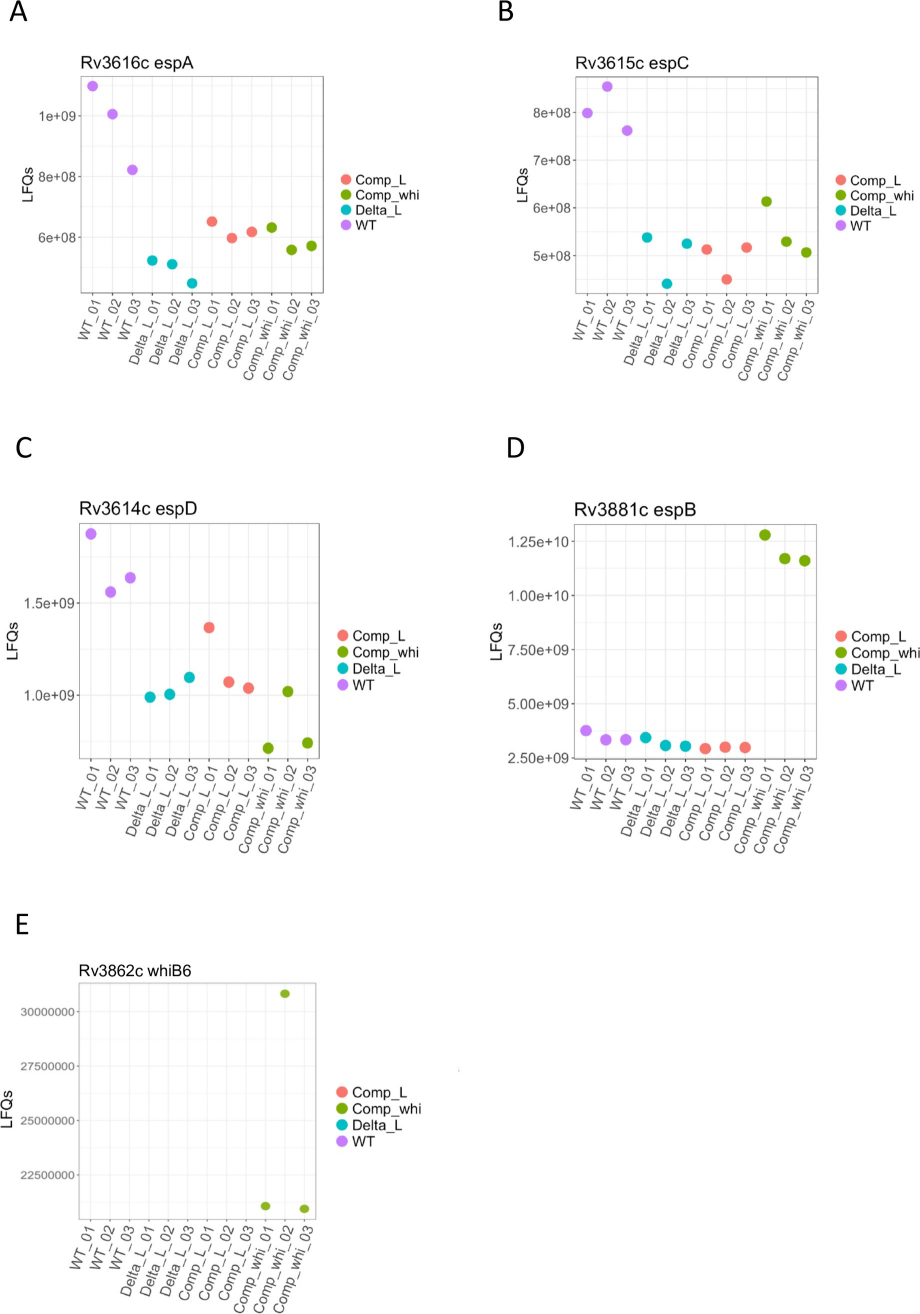
Mass spectrometry analysis of protein abundance in the Δ*espL* mutant. Total protein extracts were prepared in triplicate and subjected to Mass Spectrometry analysis. Each graph shows the abundance of the indicated protein in the three replicates in the various strains. **A)** EspA. **B)** EspC. **C)** EspD. **D)** EspB. **E)** WhiB6. WT: H37Rv. Delta_L: Δ*espL*. Comp_L: Δ*espL*/*espL* (complemented strain). Comp_whi: Δ*espL*/*whiB6* (strain expressing *whiB6 in trans*). Data are expressed as LFQs (Label Free Quantifications).

Inspired by the work of Stoop and colleagues [49], we thoroughly analyzed the proteome of the *espL* knock-out mutant and compared it to that of the wild type, of the complemented strain and of the mutant expressing *whiB6 in trans*. Results are reported in Fig 5, Fig 6, S7 Fig, S3 Table and S5 Table. As expected, EspL was detected in the wild type and in the complemented strains only (Fig 5A). On the other hand, WhiB6 levels could only be measured in the strain over-expressing *whiB6*, indicating that this transcriptional regulator is poorly expressed in wild type conditions (Fig 6E). No significant difference was noted for EspB, EspA, EspC and EspD in the proteome of Δ*espL* (Fig 6D, 6A, 6B and 6C), whilst a small though statistically valid increase in EsxA and EsxB levels was reported (Fig 5B and 5C), thus corroborating the data obtained by immunoblot (Fig 3B). The most relevant variation in protein levels was noticed for EspE, EspF and EspH. Their abundance was greatly reduced in the mutant strain and complemented to wild type levels in Δ*espL*/pGA-*espL* (Fig 5D, 5E and 5F). Proteomic data contrasted with RNA-seq and qRT-PCR results, which proved that *espE*, *espF* and *espH* mRNAs in the *espL* knock-out strain were unaltered compared to the wild type (S1 Table and S6 Fig).

EsxA, EsxB, EspB and EspE amounts increased when whiB6 was provided ectopically (Figs 5B, 5C, 6D and 5D), thus reflecting the qRT-PCR assay in Fig 4A. However, EspF and EspH levels did not reach those of H37Rv upon WhiB6 expression in Δ*espL* (Fig 5E and 5F), in contrast to their transcripts which were induced by WhiB6 (Fig 4A). These results suggest that an additional, post-transcriptional control regulates the abundance of EspF, EspH and, most likely EspE, in *M*. *tuberculosis*. In the latter case, transcriptional upregulation bypassed the destabilization provoked by lack of EspL in strain Δ*espL*/pGA-*whiB6*, thus generating increased protein abundance. This did not happen in the case of EspF (Fig 5E) and EspH (Fig 5F). The *espE* cistron is probably more efficiently translated as its gene is the first in the operon and therefore less subject to mRNA degradation. EspL contribution is more evident for EspF and EspH, as the transcriptional increase caused by WhiB6 is not sufficient to avoid the destabilizing effect on the proteins provoked by lack of EspL.

**Fig 7:**
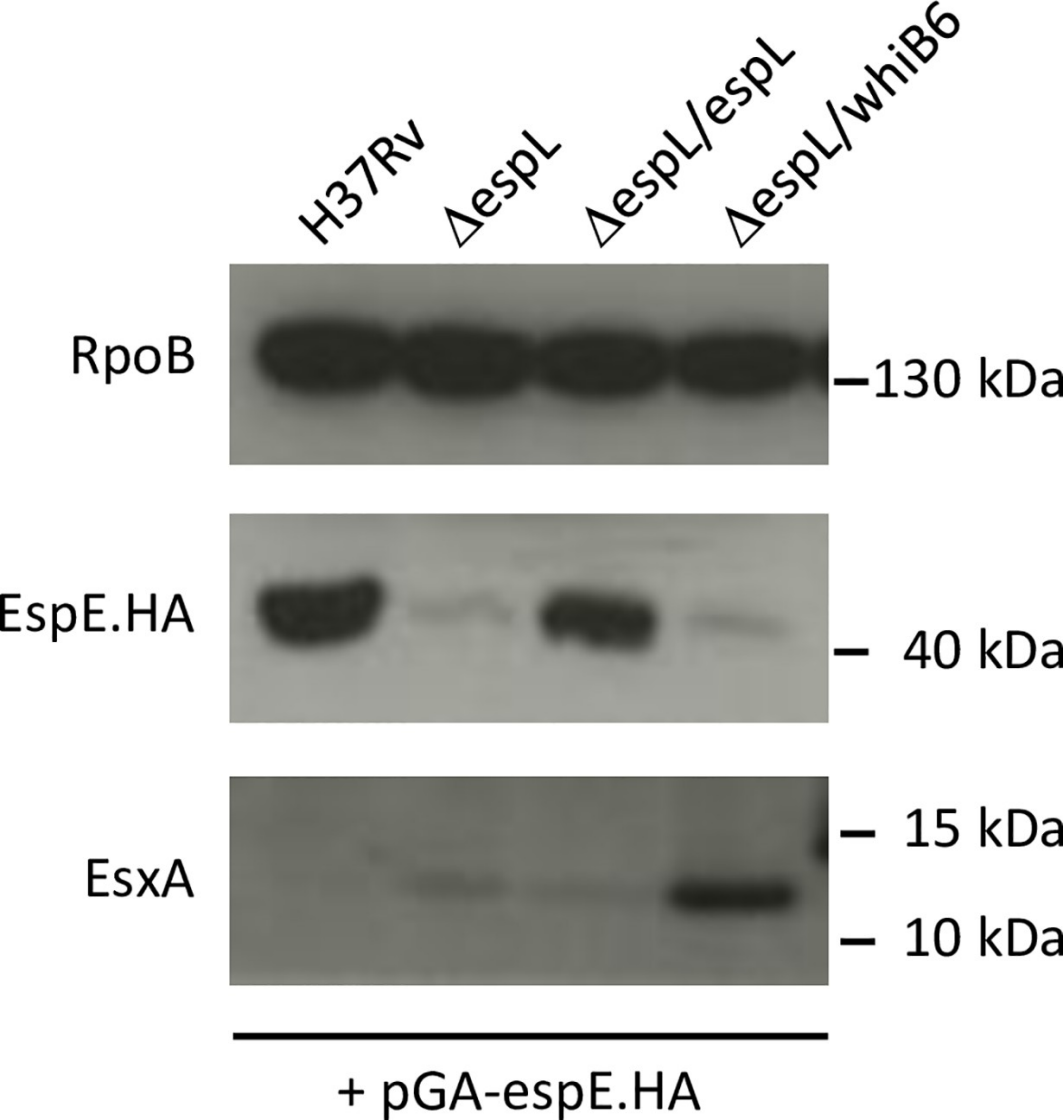
Validation of EspE levels in total cell lysates. Total cell lysates prepared from the indicated bacterial strains were analyzed by immunoblot. Note that all of the strains express HA-tagged EspE ectopically. EspE.HA was detected by immunodecoration with anti-HA antibodies. The experiment was repeated two times. One representative image is shown.

Further confirmation to these findings was obtained by constructing strains that constitutively expressed HA-tagged EspE in the H37Rv and Δ*espL* backgrounds. Complementation by *espL* and expression of *whiB6* were achieved by using the physiological *mycP1* promoter, which represents the natural promoter of *espL* according to Cortes and colleagues [37]. Expression of *espE*.HA was therefore independent from EspL and from WhiB6 as it was placed under control of the PTR promoter [39]. The wild type phenotype was restored in strain Δ*espL*/pGA-*espE*.HA + *espL*, as shown in S8A and S8B Fig, thereby confirming that expression of EspE.HA did not cause abnormal behavior. In line with what has been described before, expression of *whiB6* did not complement the lack of virulence in Δ*espL*/pGA-*espE*.HA + *whiB6* (S8B Fig) but did increase the transcriptional levels of *espE* and *esxA* (S8C Fig), the latter result mirrored by the detection of a strong signal for the EsxA protein in Fig 7. Transcription of *espE*.HA was measured by qRT-PCR and confirmed as constitutive, almost identical in all of the strains, independently of the presence or absence of EspL and WhiB6 (S8A and S8C Fig). However, immunoblot analysis demonstrated that EspE.HA amounts in Δ*espL*/pGA-*espE*.HA were dramatically reduced (Fig 7), despite the presence of the *espE*.HA transcript (S8A Fig). Conversely, Δ*espL*/pGA-*espE*.HA + *espL* (expression of *espL in trans*) produced levels of EspE.HA equal to those in H37Rv/pGA-*espE*.HA. Providing *whiB6* only (Δ*espL*/pGA-*espE*.HA + *whiB6*) did not restore the phenotype (Fig 7). Therefore, the effects mediated by EspL and WhiB6 were uncoupled here: WhiB6 was proved to act at the transcriptional level, whereas EspL was demonstrated to exert its function post-transcriptionally, presumably on protein stability.

Taken together, these results highlight a new role for EspL in stabilizing EspE, EspF and EspH protein amounts.

### EspL interacts with EspD

**Fig 8:**
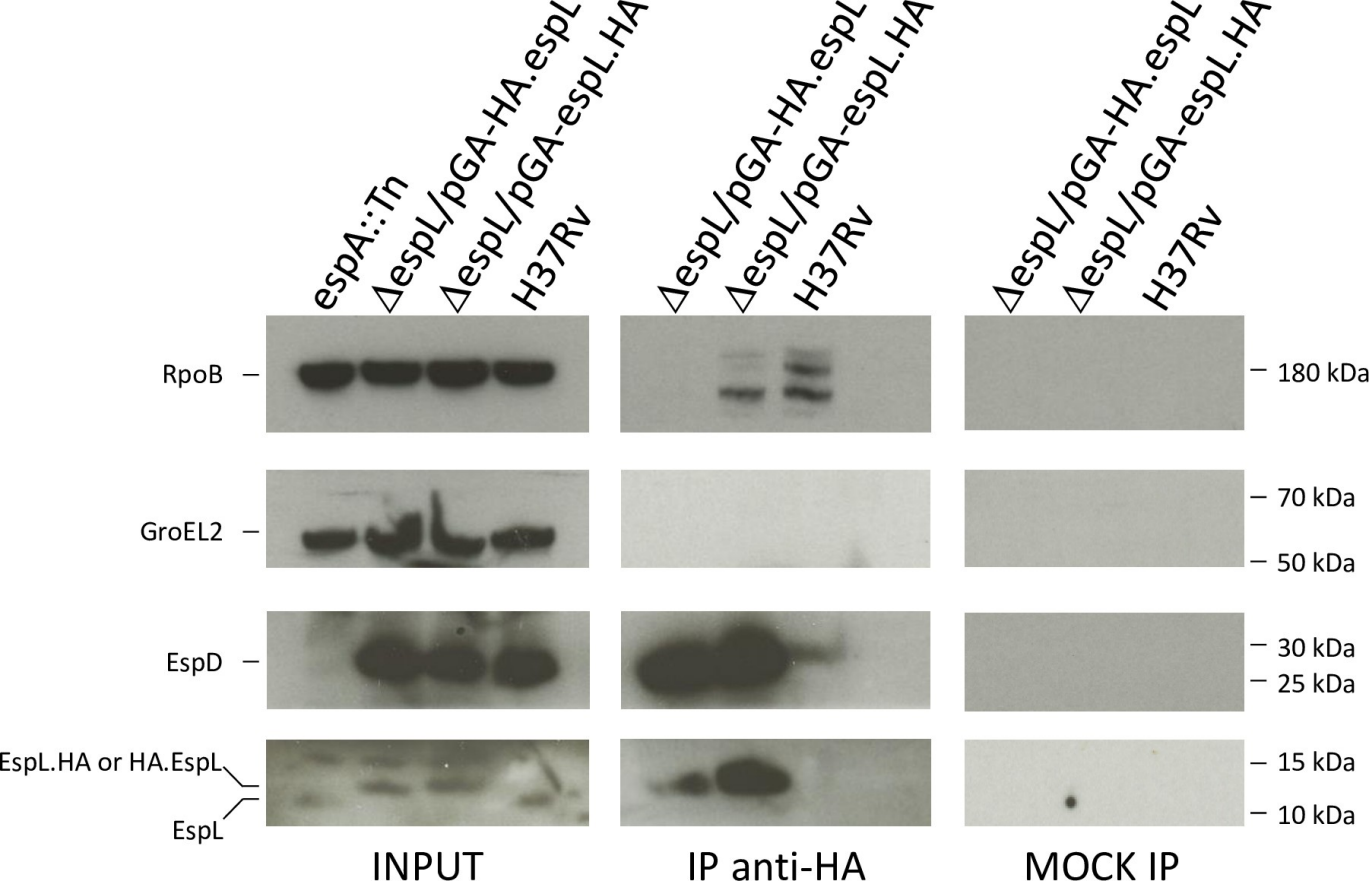
Interaction of EspL with EspD. Immunoprecipitation experiments on total protein extracts from the indicated strains were performed using anti-HA antibodies. Immunoprecipitated material was analyzed by immunoblot for detection of RpoB, GroEL2, EspL and EspD. The mock control was run in parallel without antibodies. The experiment was repeated three times. One representative image is shown.

To identify EspL interacting partners, strains carrying HA-tagged EspL were made. Expression of N- or C-terminally tagged EspL in Δ*espL* was verified by immunoblot (S9A Fig) and the ability of the modified proteins to complement the attenuation profile was checked (S9B Fig). Total cell extracts of strains Δ*espL*/pGA-*espL*.HA and Δ*espL*/pGA-HA.*espL* were employed in immunoprecipitation experiments using anti-HA antibodies. Mass spectrometry analysis of the precipitated material demonstrated that EspD was significantly enriched in the pulled-down fractions of Δ*espL*/pGA-HA.*espL* and of Δ*espL*/pGA-*espL*.HA, together with HA.EspL and EspL.HA (S6 Table). Other proteins were detected but their abundance was not increased in the immunoprecipitated samples compared to the Input and to the untagged strains H37Rv and Δ*espL* (S6 Table). A second readout, i.e. immunoblot, was exploited to validate these data independently. As shown in Fig 8, both EspD and EspL levels were highly enriched upon anti-HA immunoprecipitation in both Δ*espL*/pGA-HA.*espL* and Δ*espL*/pGA-*espL*.HA as compared to the Input control and strain H37Rv. On the other hand, RpoB and GroEL2 levels were as expected. To conclude, EspL and EspD may interact directly or be part of a multiprotein complex inside *M*. *tuberculosis* cells.

## Discussion

The data presented here demonstrate the essentiality of EspL for ESX-1-dependent virulence and for stabilizing the intracellular levels of EspE, EspF and EspH in *M*. *tuberculosis*. ESX-1-dependent secretion in Δ*espL* was severely compromised, with undetectable levels of EsxA, EsxB, EspA and EspD in the culture filtrates. Conversely, secretion of EspB was not affected, confirming previous data generated by our group [41]. In line with the secretion profile, virulence and innate cytokine production were compromised when THP-1 cells were infected by the *espL* knock-out strain. In this regard, Δ*espL* behaves like the ESX-1-null mutants ΔRD1 [13] and ΔΔRD1 [40], which fail to stimulate the innate immune response [15]. These phenotypes tally with those previously reported for an *espL* transposon mutant in a clinical isolate of the W/Beijing family, which was shown to have lost the ability to arrest phagosomal maturation [50], and with the need for *espL* in order to fully complement an *espB* mutant in *M*. *marinum* [51]. While these phenotypic traits are most likely attributable to lack of secretion of the main ESX-1 substrates, whether all of them are directly caused by lack of EspL or are mediated by EspE, EspF or EspH is currently unknown and additional research is required.

Although the mechanistic details of these functions remain unknown, a role for EspL as a chaperone protein can be proposed. Indeed, the presence of heterodimeric complexes, where one protein acts as a chaperone for the other, is not unusual in the ESX-1 system [24,52]. A direct effect of EspL on transcription of *espE*, *espF* and *espH* was ruled out by RNA-seq and further confirmed by qRT-PCR. On the other hand, proteomics identified EspE, EspF and EspH as the only proteins whose abundance was highly affected by *espL* deletion. Based on these findings, an interaction between EspL and EspE, EspF and EspH could be hypothesized. However, those proteins were not detected by mass-spectrometry and immunoblotting analysis of immunoprecipitated material from strains expressing EspL.HA or HA.EspL.

EspL-mediated stabilization of EspE, EspF and EspH levels might therefore occur by other means. Interestingly, compelling evidence was obtained for EspL interacting with EspD, which itself is known to act as a stabilizer [53], further suggesting the existence of a “chaperone complex” which contributes to regulating ESX-1 activity post-transcriptionally and/or post-translationally. Curiously, while EspD stabilizes EspA and EspC [53], EspL performs the same task on EspE, EspF and EspH, whose genes are paralogs of *espA-espC-espD* [9], although EspL and EspD are different in size and sequence.

Secretion of EspD deserves additional discussion. While EspL is necessary for secretion of the ESX-1 substrates EsxA, EsxB, EspA, EspC and for extracellular release of EspD, this was previously reported to be independent of a functional ESX-1 apparatus [53]. These observations are consistent with the findings presented here as the requirement of EspL for secretion of EspD does not imply that EspD be released through the ESX-1 system. Of note, *espL* is only present in the ESX-1 cluster and its function may serve more than one type VII secretion system and target EspD to alternative machineries, namely ESX-2, ESX-3 or ESX-4.

**Fig 9:**
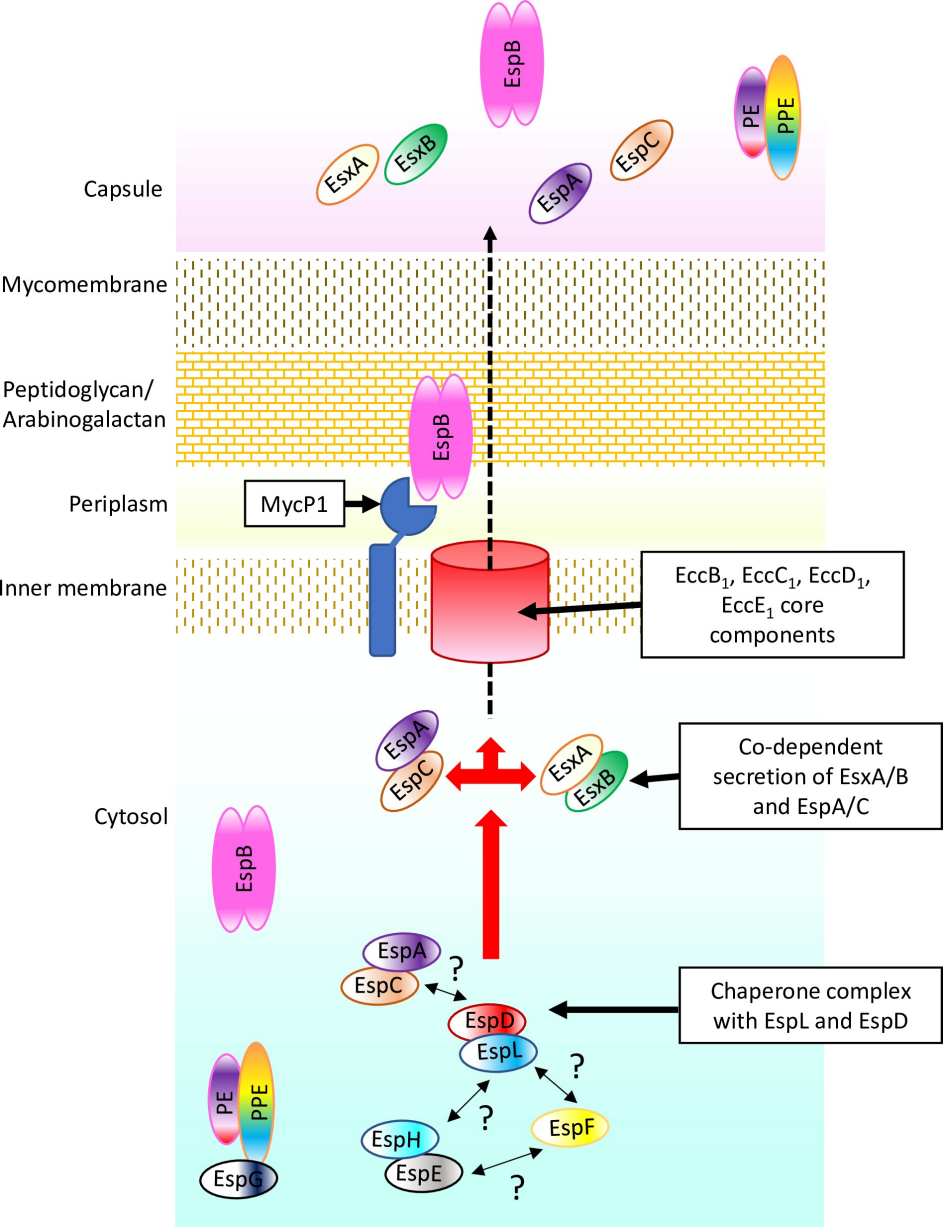
Proposed model for ESX-1-dependent secretion. In this model EspL forms a chaperone complex with EspD and this in turn stabilizes the EspA-EspC, EspE-EspH dimers and EspF. The chaperone complex may target EspA-EspC to the secretion machinery in the inner membrane, where co-dependent secretion with EsxA-EsxB takes place. Question marks indicate hypothetical protein-protein interactions. Not drawn to scale.

A model for ESX-1-dependent secretion can be proposed based on the current knowledge and on the data presented here (Fig 9). EspL forms a chaperone complex with EspD and this in turn stabilizes the EspA-EspC, EspE-EspH dimers and EspF. The chaperone complex may target EspA-EspC to the secretion machinery in the inner membrane, where co-dependent secretion with EsxA-EsxB takes place.

Another interesting finding was the discovery of *whiB6* as the only deregulated gene in the Δ*espL* transcriptome. Despite reduced expression of *whiB6*, no difference in the mRNA levels of the genes belonging to the WhiB6 putative regulon [33] was observed. This can be ascribed to the culture conditions used in these experiments, as it was reported that WhiB6 senses reducing conditions in *M*. *marinum*, and regulates transcription accordingly, thanks to its Fe-S cluster [33]. Additionally, the 3-fold deregulation of *whiB6* may not be mirrored by deregulation of its own regulon. Nonetheless, when WhiB6 was provided *in trans*, expression of most of the ESX-1 substrates or components was increased. Thereby, WhiB6 controls transcription of the ESX-1 genetic locus in *M*. *tuberculosis* too. The latter conclusion further supports the work by Solans and colleagues [35], who discovered that a single nucleotide insertion in the promoter region of *whiB6* determines the response to the transcriptional regulator PhoP and thus modulates EsxA levels.

A recent report described similar an even more pronounced deregulation of *whiB6* and of the WhiB6-dependent genes in *M*. *marinum* lacking *eccCb*_*1*_ [54]. In that case, the existence of a negative feedback loop connecting the ESX-1 core complex in the membrane to ESX-1 gene expression was postulated [54]. This is consistent with our findings in *M*. *tuberculosis* as EspL localizes mainly in the cytosol but also in the membrane fraction. Another analogy with the *eccCb*_*1*_ mutant in *M*. *marinum* lies in the EspE and EspF protein levels, which also seem to be subjected to post-transcriptional control [54]. Of note, we demonstrated that virulence cannot be restored in Δ*espL* by expression of WhiB6 *in trans*.

Altogether, our data indicate an important role for EspL in *M*. *tuberculosis* pathogenesis and encourage further investigations into the contributions of EspE, EspF and EspH to virulence and to ESX-1-dependent secretion. EspF was previously shown to reduce *M*. *tuberculosis* virulence in the mouse model when deleted [40]. EspH was recently identified as necessary for secretion of EspE and EspF in *M*. *marinum* and capable of binding EspE, thus acting as a potential chaperone [55]. Furthermore, essentiality of EspH for phagocytic infection as well as for granuloma formation in the zebrafish larvae model was reported [55]. On the contrary, little is known about the role of EspE. Conservation of the genes for *espH*, *espD* and *espL*, in the greatly down-sized genome of *M*. *leprae* [56] suggests a conserved function and this justifies future investigation.

## Materials and methods

### Bacterial strains and culture conditions

*Mycobacterium tuberculosis* strains (described in S7 Table) were grown at 37°C in 7H9 medium (Difco) supplemented with 0.2% glycerol, 0.05% Tween 80 and 10% albumin-dextrose-catalase (ADC, Middlebrook) or on 7H10 plates supplemented with 0.5% glycerol and 10% oleic acid-albumin-dextrose-catalase (OADC, Middlebrook). Sauton’s liquid medium was used for culture filtrate analysis. Streptomycin (20 μg/ml), kanamycin (20 μg/ml), hygromycin (50 μg/ml) or 2.5% sucrose were added when necessary. Experiments involving *M*. *tuberculosis* were performed in a Biosafety Level 3 (BSL3) laboratory, according to the national and international guidelines (Authorization number A070027/3). For cloning purposes, One Shot TOP10 chemically competent *Escherichia coli* (Invitrogen) were grown in Luria–Bertani (LB) broth or on LB agar with hygromycin (200 μg/ml), kanamycin (50 μg/ml) or spectinomycin (25 μg/ml).

### Reagents, plasmid vectors and oligonucleotides

Chemical reagents were obtained from Sigma-Aldrich, unless otherwise stated. Restriction and modification enzymes were purchased from New England Biolabs. Plasmid vectors are described in S8 Table. Oligonucleotides were synthesized by Microsynth. Sequences are available upon request.

### Mutant construction

One kb up- and downstream regions of the *espL* gene were PCR-amplified, ligated in-frame with the AvrII site and cloned into the PacI and AscI sites of pJG1100 [38], resulting in the suicide vector pJG1100-espL-UP/DOWN. The complementing plasmid pGA44-espL was constructed by cloning the *espL* gene into vector pGA44 [39], under control of the PTR promoter. Deletion of the full-length *espL* gene was achieved by homologous recombination using plasmid pJG1100-espL-UP/DOWN. After transformation of *M*. *tuberculosis* H37Rv, the first recombination event was selected on 7H10 plates, supplemented with hygromycin and kanamycin. Colonies were screened by colony PCR. Two clones that had undergone homologous recombination were grown in liquid 7H9 medium with no antibiotics, in order to promote the second recombination event and plasmid excision. Selection of the recombinant clones was performed by plating the bacteria on 7H10 plates supplemented with sucrose. The resulting colonies were tested by PCR to confirm deletion of *espL* from its native locus and further validated by whole genome sequencing.

### *In vitro* growth curves

*M*. *tuberculosis* strains were grown to mid-logarithmic phase and then diluted to an optical density at 600 nm (OD_600_) of 0.05 in 7H9 medium. OD_600_ was recorded at different time points to obtain the growth curves.

### Genomic DNA preparation and whole genome sequencing

*M*. *tuberculosis* genomic DNA was extracted as previously described [57]. Libraries were prepared using the Kapa LTP Library Prep kit (Kapa Biosystems) according to the manufacturer’s recommendations. Cluster generation was performed using the Illumina TruSeq SR Cluster Kit v4 reagents and sequenced on the Illumina HiSeq 2500 using TruSeq SBS Kit v4 reagents. Sequencing data were demultiplexed using the bcl2fastq Conversion Software (v. 2.20, Illumina, San Diego, California, USA). Raw reads were adapter- and quality-trimmed with Trimmomatic v0.33 [58]. The quality settings were “SLIDINGWINDOW:5:15 MINLEN:40”. Preprocessed reads were mapped onto the *M*. *tuberculosis* H37Rv reference genome sequence (RefSeq NC_000962.3) with Bowtie2 v2.2.5 [59]. SNP calling was done using VarScan v2.3.9 [60]. To avoid false-positive SNP calls the following cutoffs were applied: minimum overall coverage of ten non-duplicated reads, minimum of five non-duplicated reads supporting the SNP, mapping quality score >8, base quality score >15, and a SNP frequency above 80%. All SNPs were manually checked by visualizing the corresponding read alignments. Sequencing data have been deposited to the Sequence Read Archive (SRA) under accession number SRP158673.

### RNA preparation, reverse-transcription and quantitative polymerase chain reaction (RT-qPCR)

*M*. *tuberculosis* cultures were grown to OD_600_ of 0.3–0.4, harvested by centrifugation, pellets were resuspended in TRIzol Reagent (ThermoFisher) and stored at -80°C until further processing. Total RNA was extracted by bead-beating as previously described [39]. Integrity of RNA was checked by agarose gel electrophoresis, purity and amount of RNA were assessed using a Nanodrop instrument and Qubit Fluorometric Quantitation (ThermoFisher), respectively. SuperScript III First-Strand Synthesis System (Invitrogen) was used to generate randomly primed cDNA from 1 μg of RNA, according to the manufacturer’s recommendations. qPCR reactions were performed on an ABI 7900HT instrument, using Power SybrGreen PCR Master Mix (Applied Biosystems), according to the manufacturer’s instructions. The housekeeping gene *sigA* was used for normalization.

### RNA-seq: Library preparation, high-throughput sequencing and analysis

RNA was extracted from biological duplicates as described above. RNA-seq libraries were prepared from 1 μg of total RNA. The RNA samples were depleted of r-RNAs with the Illumina Ribo-Zero rRNA Removal Kit (Gram-Positive Bacteria) then used to generate sequencing libraries with the Illumina TruSeq Stranded mRNA reagents, omitting the polyA selection step (Illumina, San Diego, California, USA). Cluster generation was performed with the resulting libraries using the Illumina TruSeq SR Cluster Kit v4 reagents and sequenced on the Illumina HiSeq 2500 using TruSeq SBS Kit v4 reagents. Sequencing data were demultiplexed using the bcl2fastq Conversion Software (v. 2.20, Illumina, San Diego, California, USA).

Reads were processed and mapped to the reference genome sequence as described above. Counting reads over features was done with featureCounts [61] from the Subread package v1.4.6. Annotation was taken from TubercuList release R27 (https://mycobrowser.epfl.ch/releases). Differential gene expression analysis was done using DESeq2 [62]. RNA-seq data have been deposited to the Gene Expression Omnibus (GEO) repository under accession number GSE118994.

### Mass spectrometry analysis of total protein extracts and culture filtrates

*M*. *tuberculosis* cells, grown to mid-exponential phase in 15 ml cultures, were pelleted by centrifugation, washed once in PBS (Phosphate Buffered Saline) supplemented with 0.05% Tween 80 and the pellets stored at -80°C until further use. Total lysates were obtained by sonication in lysis buffer (100 mM Tris pH 8, 2% SDS, cOmplete mini EDTA free Roche) and boiled at 100°C for 1 h. Proteins were quantified by using the Pierce BCA Protein Assay kit and 30 μg were submitted for mass spectrometry analysis. For the analysis of the culture filtrates, *M*. *tuberculosis* cultures were grown in 7H9 to mid-logarithmic phase. The culture medium was then replaced by Sauton’s supplemented with 0.05% Tween 80 and growth was continued for 3 d. Finally, bacteria were pelleted and resuspended in Sauton’s medium without Tween 80 for collection of the culture filtrates. These were filtered through 0.22 μm Steriflip Millipore Express Plus Membranes (Millipore), concentrated 100x using Amicon Ultracel-3K centrifugal filters (Millipore), quantified by using the Pierce BCA Protein Assay kit and 30 μg were submitted for mass spectrometry analysis.

Each sample was digested by Filter Aided Sample Preparation (FASP) [63] with minor modifications. Dithiothreitol (DTT) was replaced by Tris(2-carboxyethyl)phosphine (TCEP) as reducing agent and Iodoacetamide by Chloracetamide as alkylating agent. A combined proteolytic digestion was performed using Endoproteinase Lys-C and Trypsin. Acidified peptides were desalted on C18 StageTips [64] and dried down by vacuum centrifugation. For LC MS/MS analysis, peptides were resuspended and separated by reversed-phase chromatography on a Dionex Ultimate 3000 RSLC nanoUPLC system in-line connected with an Orbitrap Fusion Lumos Mass-Spectrometer (Thermo Fischer Scientific). Database search was performed using MaxQuant 1.6.0.1 [65] against the TubercuListR27 database (http://tuberculist.epfl.ch/). Carbamidomethylation was set as fixed modification, whereas oxidation (M), phosphorylation (S,T,Y) and acetylation (Protein N-term) were considered as variable modifications. Label Free Quantification (MaxLFQ) was performed by MaxQuant using the standard settings [66]. Perseus [67] was used to highlight differentially quantified proteins. Reverse proteins, contaminants and proteins only identified by sites were filtered out. Biological replicates were grouped together and protein groups containing a minimum of two LFQ values in at least one group were conserved. Empty values were imputed with random numbers from a normal distribution. The average LFQ values for the different proteins in the different strains were obtained from columns DM-DO, DP-DR and DS-DU in S2 Table, from columns EK-EM, EN-EP, EQ-ES, ET-EV in S3 Table. The difference between these numbers represents the “difference in protein abundance”. Significant hits were determined by a volcano plot-based strategy, combining t test p-values with ratio information [68]. Significance curves in the volcano plot corresponding to a SO value of 0.5 and 0.05 FDR (for culture filtrates) and to a SO value of 0.1 and 0.05 FDR (for cell lysates) were determined by a permutation-based method. Further graphical displays were generated using homemade programs written in R [69]. Raw data obtained from mass spectrometry experiments have been deposited to the ProteomeXchange Consortium via the PRIDE partner repository with the dataset identifiers PXD010929 (total cell lysate) and PXD011466 (culture filtrate).

### Immunoblot analysis of bacterial lysates and culture filtrates

Culture filtrates were obtained as described above for mass spectrometry analysis, quantified by using the Qubit Fluorometric Quantitation device (ThermoFisher) and loaded on SDS-PAGE 12–15% NuPAGE gels (Invitrogen) for immunoblot analyses. Bacterial pellets were washed once in Tris-Buffered Saline (TBS, 20 mM Tris-HCl pH 7.5, 150 mM NaCl) and stored at -80°C until further processing. Cells were sonicated in TBS supplemented with a protease inhibitor tablet (cOmplete mini EDTA free, Roche) for 15 min and the protein solution was then sterilized by filtration through a 0.22 μm filter (Pall Life Sciences) to remove residual intact cells. Protein samples were quantified using Qubit. Equal amounts of protein preparations (10 μg for cell lysates, 15–20 μg for culture filtrates) were loaded on SDS-PAGE 12–15% NuPAGE gels (Invitrogen) and transferred onto PVDF membranes using a semidry electrophoresis transfer apparatus (Invitrogen). Membranes were incubated in TBS-Tween blocking buffer (25 mM Tris pH 7.5, 150 mM NaCl, 0.05% Tween 20) with 5% w/v skimmed milk powder for 2h at 4°C prior to overnight incubation with primary antibody. Membranes were washed in TBS-Tween three times at room temperature, and then incubated with secondary antibody for 3 h before washing again. Signals were detected using Chemiluminescent Peroxidase Substrate 1 (Sigma-Aldrich).

Polyclonal anti-EspL, anti-EspB, anti-EspA [53], anti-EspD [53] antibodies were produced by Dr. Ida Rosenkrands (Statens Serum Institut, Copenhagen, Denmark). Monoclonal anti-RpoB antibodies were purchased from NeoClone, polyclonal anti-EsxB antibodies from Abcam, monoclonal anti-HA antibodies conjugated to Horseradish Peroxidase (HRP) from Cell Signaling. Polyclonal anti-Rv3852 antibodies were generated by Eurogentec [42]. The following reagents were obtained through BEI Resources, NIAID, NIH: monoclonal anti-Antigen 85, monoclonal anti-GroEL2 and polyclonal anti-EsxA antibodies.

### Fractionation of total bacterial lysate

Cell fractions were obtained as described previously [42]. Briefly, *M*. *tuberculosis* was grown in Sauton’s medium with 0.05% Tween 80 to mid-exponential phase, cells were collected by centrifugation, supernatants were filtered through 0.22 μm Steriflip Millipore Express Plus Membranes (Millipore) and concentrated 100x using Amicon Ultracel-3K centrifugal filters (Millipore) to obtain the culture filtrate fraction. The pellet was treated with 0.25% Genapol-X080 for 30 min at room temperature, followed by centrifugation at 14,000 g for 10 min. The proteins in the resulting supernatant were precipitated with Trichloroacetic acid (TCA), yielding the capsular fraction. The remaining pellet was subjected to sonication to break the cells, sterilized by filtration through a 0.22 μm filter (Pall Life Sciences) followed by ultra-centrifugation at 45,000 rpm for 1h at 4°C. The supernatant contained the cytosolic fraction, while the pellet was enriched with membrane proteins. Analysis of the protein fractions was carried out by immunoblot as described above.

### Co-immunoprecipitation experiments

*M*. *tuberculosis* cells in 30 ml cultures were pelleted by centrifugation, washed once in PBS (Phosphate Buffered Saline) supplemented with 0.05% Tween 80 and the pellets stored at -80°C until further use. Total lysates were obtained by sonication in TBS-T (25 mM Tris pH 7.5, 150 mM NaCl, 0.05% Tween 20), followed by filtration through 0.22 μm filters (Pall Life Sciences). Fifty microliters of Monoclonal Anti-HA Agarose Antibody beads (Sigma-Aldrich) were incubated with approximately 1 mg of bacterial extract in Spin-X centrifuge tubes (Costar) for 4 h at 4°C on an orbital shaker. The resin was washed four times in PBS and the immunoprecipitated material was eluted from the beads in PBS-SDS sample buffer (100 mM Tris HCl pH 6.8, 200 mM dithiothreitol, 4% SDS, 0.2% bromophenol blue, 20% glycerol) during a 5 min incubation at 95°C. Immunoprecipitated proteins were analyzed either by mass spectrometry as described [24] or by immunoblot. A mock (no antibody) control was run in parallel with agarose beads only.

### Cell cultures and infection with *M*. *tuberculosis* strains

THP-1 cells (ATCC-TIB202, LGC Standards GmbH, Germany) were cultured in RPMI1640 (Life Technologies) supplemented with 10% (v/v) Fetal Calf Serum (Life Technologies) and 1% sodium pyruvate (Life Technologies). Cells were differentiated in 96- or 12-well plates by addition of 4 nM phorbol 12-myristate 13-acetate (PMA) for 24 h at 37°C in 5% CO_2_. Differentiated cells were then infected with *M*. *tuberculosis* as follows. Bacteria were grown to exponential phase (OD_600_ between 0.4 and 0.8), washed once in 7H9 medium, resuspended in 7H9 to an OD_600_ of 1, equivalent to 3 x 10^8^ bacteria/ml. The required volume of bacterial suspension was then added to RPMI1640 for infection of human THP-1 cells at the multiplicity of infection (MOI) reported in the text. Plates were sealed with gas-permeable sealing film and incubated at 37°C under 5% CO_2_. Intracellular bacteria were released by the infected cells by addition of 0.5% Triton-X. The suspension was serially diluted in 7H9 and plated on 7H10 plates. Colony forming units (CFU) were counted after incubation at 37°C for 4–5 weeks. PrestoBlue Assay (Thermofisher) to evaluate cell viability was performed according to the manufacturer’s instructions. Fluorescence was measured using a Tecan Infinite M200 microplate reader.

### ELISA assays

Cell culture supernatants from infections in 96-well plates were removed from infected cells 24 h post-infection. Supernatants were filtered through NANOSEP centrifugal devices (Pall Life Sciences) and assayed for human IL-1β (BD Biosciences) according to the manufacturer’s instructions.

### Quantitative polymerase chain reaction (qPCR) analysis of cytokine expression

RNA from infected cells in the 12-well format was extracted by using Qiagen RNeasy kit according to the manufacturer’s instructions 24 h post-infection and reverse-transcribed using the RevertAid First Strand cDNA Synthesis kit (Fermentas). Quantitative PCR analysis was performed on an ABI 7900HT instrument. All gene expression data are presented as relative expression to GAPDH.

### Statistical analysis

Statistical analyses were performed in GraphPad PRISM by one-way or two-way ANOVA followed by Tukey’s multiple comparison test.

## Supporting information

S1 Table This Table contains the results of RNA-seq experiments performed on the wild type strain H37Rv and on the mutant strain ΔespL. (XLSX)

S2 Table This Table contains the results of the proteomics experiments performed on the culture filtrates of the wild type strain H37Rv, of the mutant strain ΔespL, of the complemented mutant ΔespL/espL. (XLSX)

S3 Table This Table contains the results of the proteomics experiments performed on total cell lysates of the wild type strain H37Rv, of the mutant strain ΔespL, of the complemented mutant ΔespL/espL and of the mutant transformed with a plasmid overexpressing whiB6. (XLSX)

S4 Table Results of mass spectrometry experiments_Secretome. This Table represents an excerpt from S2 Table and lists ESX-1 secreted proteins as well as control proteins.
(XLSX)

S5 Table Results of mass spectrometry experiments_Total cell lysate. This Table represents an excerpt from S3 Table and lists ESX-1 secreted proteins and components as well as control proteins.
(XLSX)

S6 Table Immunoprecipitation experiment analyzed by mass spectrometry. This Table contains the results of the anti-HA immunoprecipitation experiment.
(PDF)

S7 Table Bacterial strains used in this study. This table lists the bacterial strains used in this study.
(PDF)

S8 Table Plasmids used in this study. This table lists the plasmids used in this study.
(PDF)

S1 Fig Construction of Δ*espL* mutant. **A)** Schematic representation of the H37Rv genomic region that encompasses the *espL* gene. The 5’-ends of the mRNAs detected by Cortes and colleagues [37] are indicated by bent arrows. **B)** Construction of the Δ*espL* mutant by allelic exchange. An unmarked deletion was introduced into the *espL* native locus.
(PDF)

S2 Fig Phenotypic analysis of Δ*espL* mutant. **A)** Growth curves obtained by measuring the optical density at 600 nm of the different strains grown in 7H9 medium at 37°C with shaking. **B)** Uptake of various bacterial strains by THP-1 cells. THP-1 cells were infected at multiplicity of infection (MOI) of 1. The number of intracellular bacteria was evaluated by CFU 3 h post-infection. Data were expressed as the mean and SD of two independent replicates. ΔΔRD1 carries a deletion of the extended ESX-1 locus. ns, not significant in one-way ANOVA followed by Tukey’s multiple comparison test.
(PDF)

S3 Fig Immunoblot analysis of EspB secretion. **A)** Total cell lysates prepared from the indicated bacterial strains were analyzed by immunoblot. Membranes were probed for EsxA, EspB and GroEL2. **B)** Culture filtrates were analyzed as described for the total cell lysates. Antigen 85 (Ag85) represents the loading control. The experiment was repeated two times. One representative image is shown.
(PDF)

S4 Fig Mass spectrometry analysis of the secretome of the Δ*espL* mutant. Volcano plot representation of the secretome comparison between Δ*espL* mutant and wild type strain. Blue lines indicate an FDR of 0.05 with a S0 = 0.5. Red points represent ESX-1 substrates.
(PDF)

S5 Fig Localization of EspL in subcellular fractions. The culture filtrate, capsular, membrane and cytosolic fractions were analyzed by immunoblot. Membranes were probed for RpoB (cytosolic control), Rv3852 (membrane control), EsxB (culture filtrate control) and EspL.
(PDF)

S6 Fig Validation of RNA-seq results by qRT-PCR. qRT-PCR analysis was performed on total RNA extracted from the indicated strains. Expression levels of the various genes were obtained from two independent replicates, normalized to the housekeeping gene *sigA* and expressed as relative to H37Rv. **, *p* < 0.005. ns, not significant in two-way ANOVA followed by Tukey’s multiple comparison test.
(PDF)

S7 Fig Mass spectrometry analysis of the total proteome of the Δ*espL* mutant. Volcano plot representation of the total proteome comparison between Δ*espL* mutant and wild type strain. Blue lines indicate an FDR of 0.05 with a S0 = 0.1. Red points represent ESX-1 substrates that were found to be underrepresented in Δ*espL*. Green points represent EsxA and EsxB, which were more abundant in Δ*espL* compared to the wild type strain.
(PDF)

S8 Fig Expression of EspE.HA in H37Rv and in Δ*espL* mutant. **A)** qRT-PCR analysis of *espL*, *whiB6*, *esxA*, *espE* and *espE*.HA gene expression levels in different strains. Data were obtained from two independent replicates, normalized to the housekeeping gene *sigA* and expressed as relative to H37Rv/pGA-*espE*.HA. *, *p* < 0.05. **, *p* < 0.005. ns, not significant in two-way ANOVA followed by Tukey’s multiple comparison test. **B)** Virulence of Δ*espL* mutant expressing *whiB6 in trans* compared to H37Rv, Δ*espL* and complemented strain in the THP-1 infection model. THP-1 cells were infected at multiplicity of infection (MOI) of 5. Note that all of the strains, except ΔΔRD1, express *espE*.HA. ΔΔRD1 carries a deletion of the extended ESX-1 locus. Fluorescence measurements directly correlate with THP-1 viability. Data were expressed as the mean and standard deviation (SD) of four independent replicates. NI: not infected control. ****, *p* < 0.0001. ns, not significant in one-way ANOVA followed by Tukey’s multiple comparison test. **C)** qRT-PCR analysis of the expression levels of the indicated genes in different strains, upon ectopic expression of *whiB6*. Data were obtained from two independent replicates, normalized to the housekeeping gene *sigA* and expressed as relative to H37Rv/pGA-*espE*.HA. ****, *p* < 0.0001. ns, not significant in two-way ANOVA followed by Tukey’s multiple comparison test.
(PDF)

S9 Fig Validation of the expression of HA-tagged EspL and virulence analysis. **A)** Immunoblot showing expression of EspL.HA and HA.EspL in total protein extracts from two independent clones (1 and 2) obtained upon transformation of Δ*espL* with a plasmid encoding *espL*.HA or HA.*espL*, respectively. Protein extract from *espC*::*Tn*/pMD*espAC*_*HA*_*D* [24] was used as a control. **B)** Virulence of Δ*espL* mutant complemented by *espL*.HA or by HA.*espL* compared to H37Rv, Δ*espL* and complemented strain in the THP-1 infection model. ΔΔRD1 carries a deletion of the extended ESX-1 locus. THP-1 cells were infected at multiplicity of infection (MOI) of 5. Fluorescence measurements directly correlate with THP-1 viability. Data were expressed as the mean and standard deviation (SD) of four independent replicates. NI: not infected control. ****, *p* < 0.0001. ns, not significant in one-way ANOVA followed by Tukey’s multiple comparison test.
(PDF)
