# Supplementary material for: EspL is essential for virulence and stabilizes EspE, EspF and EspH levels in Mycobacterium tuberculosis
Source: PLoS Pathog. 2018 Dec 20;14(12):e1007491. doi: 10.1371/journal.ppat.1007491 (PMC6319747; doi:10.1371/journal.ppat.1007491)
Supplement: S6 Table — This Table contains the results of the anti-HA immunoprecipitation experiment. (PDF) [file ppat.1007491.s006.pdf]

S6 Table. Immunoprecipitation experiment analyzed by mass spectrometry.

Protein	MW (kDa)	Counts Input Δ*espL*/HA.*espL*	Counts IP H37Rv	Counts IP Δ*espL*	Counts IP Δ*espL*/*espL*.HA	Counts IP Δ*espL*/HA.*espL*
GroEL2	57	121	16	48	58	45
DnaK	67	19	8	8	13	11
**EspL.HA or HA.EspL**	**14**	**3**	**4**	**11**	**125**	**187**
RpoB	129	3	19	5	5	2
RpoC	147	4	4	0	18	2
SigA	58	3	27	11	16	14
Tuf	44	11	0	20	8	8
mIHF	21	10	0	9	3	0
GyrA	92	1	5	11	0	0
RplK	15	3	0	6	0	2
**EspD**	**20**	**0**	**5**	**0**	**11**	**35**
EsxB	11	5	0	0	5	0

The Table reports peptide counts for the indicated proteins in the samples obtained upon immunoprecipitation (IP) with anti-HA antibodies. The total protein extract (Input) was used as a control. EspL.HA, HA.EspL and EspD are indicated in bold.
MW: molecular weight. H37Rv: wild type strain. Δ*espL*: *espL* knock-out mutant. Δ*espL*/*espL*.HA: *espL* knock-out mutant complemented by HA-tagged *espL* (HA at C-terminus). Δ*espL*/HA.*espL*: *espL* knock-out mutant complemented by HA-tagged *espL* (HA at N-terminus).
