# Supplementary material for: EspL is essential for virulence and stabilizes EspE, EspF and EspH levels in Mycobacterium tuberculosis
Source: PLoS Pathog. 2018 Dec 20;14(12):e1007491. doi: 10.1371/journal.ppat.1007491 (PMC6319747; doi:10.1371/journal.ppat.1007491)
Supplement: S7 Table — This table lists the bacterial strains used in this study. (PDF) [file ppat.1007491.s007.pdf]

S7 Table. Bacterial strains used in this study.

Bacterial species	Strain name	Description or genotype	Reference
M. tuberculosis	H37Rv	Wild type strain	[2]
	H37Rv/pGA44	H37Rv transformed with empty vector pGA44. Plasmid integrated at L5 *attB* site.	This study
	H37Rv/pGA-*espE*.HA	H37Rv expressing HA-tagged EspE. Plasmid integrated at L5 *attB* site.	This study
	ΔΔRD1	H37Rv background. Deletion of extended ESX-1 locus.	[3]
	Δ*espL*	H37Rv background. Deletion of *espL*.	This study
	Δ*espL*/pGA44	*espL* knockout transformed with empty vector pGA44. Plasmid integrated at L5 *attB* site.	This study
	Δ*espL*/pGA-*espL*	*espL* knockout complemented by *espL* expressed *in trans*.	This study
	Δ*espL*/pGA-*whiB6*	*espL* knockout transformed with vector expressing *whiB6*.	This study
	Δ*espL*/pGA-*espL*.HA	*espL* knockout complemented by *espL*.HA expressed *in trans*.	This study
	Δ*espL*/pGA-HA.*espL*	*espL* knockout complemented by HA.*espL* expressed *in trans*.	This study
	Δ*espL*/pGA-*espE*.HA	*espL* knockout transformed with vector expressing *espE*.HA.	This study
	Δ*espL*/pGA-*espE*.HA+*pmycP1-espL*	*espL* knockout transformed with vector expressing *espE*.HA and complemented by *espL* expressed *in trans*.	This study
	Δ*espL*/pGA-*espE*.HA+*pmycP1-whiB6*	*espL* knockout transformed with vector expressing *espE*.HA and *whiB6*.	This study

	espA::Tn	Erdman background. Transposon insertion in *espA*.	[4]
	espC::Tn/pMD*espAC$_{HA}$D*	Erdman background. Transposon insertion in *espC*. Complemented by pMD31-derived vector carrying *espA-espC-espD*. EspC is HA-tagged.	[5]
	Δ*espB*	H37Rv background. Deletion of *espB*.	This study
	Erdman	Wild type strain	[4]
	5' Tn::pe35	Erdman background. Transposon insertion 102 bp upstream of *pe35* start codon	[4]
E. coli	TOP10	F- *mcrA* Δ(*mrr-hsd*RMS-*mcr*BC) Φ80*lac*ZΔM15 Δ *lac*X74 *rec*A1 *ara*D139 Δ(*araleu*)7697 *gal*U *gal*K *rps*L (StrR) *end*A1 *nup*G	Invitrogen

Bibliography

1. Cortes T, Schubert OT, Rose G, Arnvig KB, Comas I, Aebersold R, et al. Genome-wide Mapping of Transcriptional Start Sites Defines an Extensive Leaderless Transcriptome in Mycobacterium tuberculosis. Cell Rep. 2013;5: 1121–1131. doi:10.1016/j.celrep.2013.10.031
2. Cole ST, Brosch R, Parkhill J, Garnier T, Churcher C, Harris D, et al. Deciphering the biology of Mycobacterium tuberculosis from the complete genome sequence. Nature. 1998;393: 537–544. doi:10.1038/31159
3. Bottai D, Majlessi L, Simeone R, Frigui W, Laurent C, Lenormand P, et al. ESAT-6 secretion-independent impact of ESX-1 genes espF and espG1 on virulence of Mycobacterium tuberculosis. J Infect Dis. 2011;203: 1155–1164. doi:10.1093/infdis/jiq089
4. Chen JM, Boy-Röttger S, Dhar N, Sweeney N, Buxton RS, Pojer F, et al. EspD is critical for the virulence-mediating ESX-1 secretion system in Mycobacterium tuberculosis. J Bacteriol. 2012;194: 884–893. doi:10.1128/JB.06417-11
5. Lou Y, Rybniker J, Sala C, Cole ST. EspC forms a filamentous structure in the cell envelope of *M ycobacterium tuberculosis* and impacts ESX-1 secretion: Filamentous structure formation by EspC. Mol Microbiol. 2017;103: 26–38. doi:10.1111/mmi.13575
6. Gomez JE, Bishai WR. whmD is an essential mycobacterial gene required for proper septation and cell division. Proc Natl Acad Sci U S A. 2000;97: 8554–8559. doi:10.1073/pnas.140225297
7. Kolly GS, Boldrin F, Sala C, Dhar N, Hartkoorn RC, Ventura M, et al. Assessing the essentiality of the decaprenyl-phospho- D -arabinofuranose pathway in *M ycobacterium tuberculosis* using conditional mutants: Druggability of the *M. tuberculosis* DPA pathway. Mol Microbiol. 2014;92: 194–211. doi:10.1111/mmi.12546
