# Supplementary material for: EspL is essential for virulence and stabilizes EspE, EspF and EspH levels in Mycobacterium tuberculosis
Source: PLoS Pathog. 2018 Dec 20;14(12):e1007491. doi: 10.1371/journal.ppat.1007491 (PMC6319747; doi:10.1371/journal.ppat.1007491)
Supplement: S1 Fig — A) Schematic representation of the H37Rv genomic region that encompasses the espL gene. The 5’-ends of the mRNAs detected by Cortes and colleagues [37] are indicated by bent arrows. B) Construction of the ΔespL mutant by allelic exchange. An unmarked deletion was introduced into the espL native locus. (PDF) [file ppat.1007491.s009.pdf]

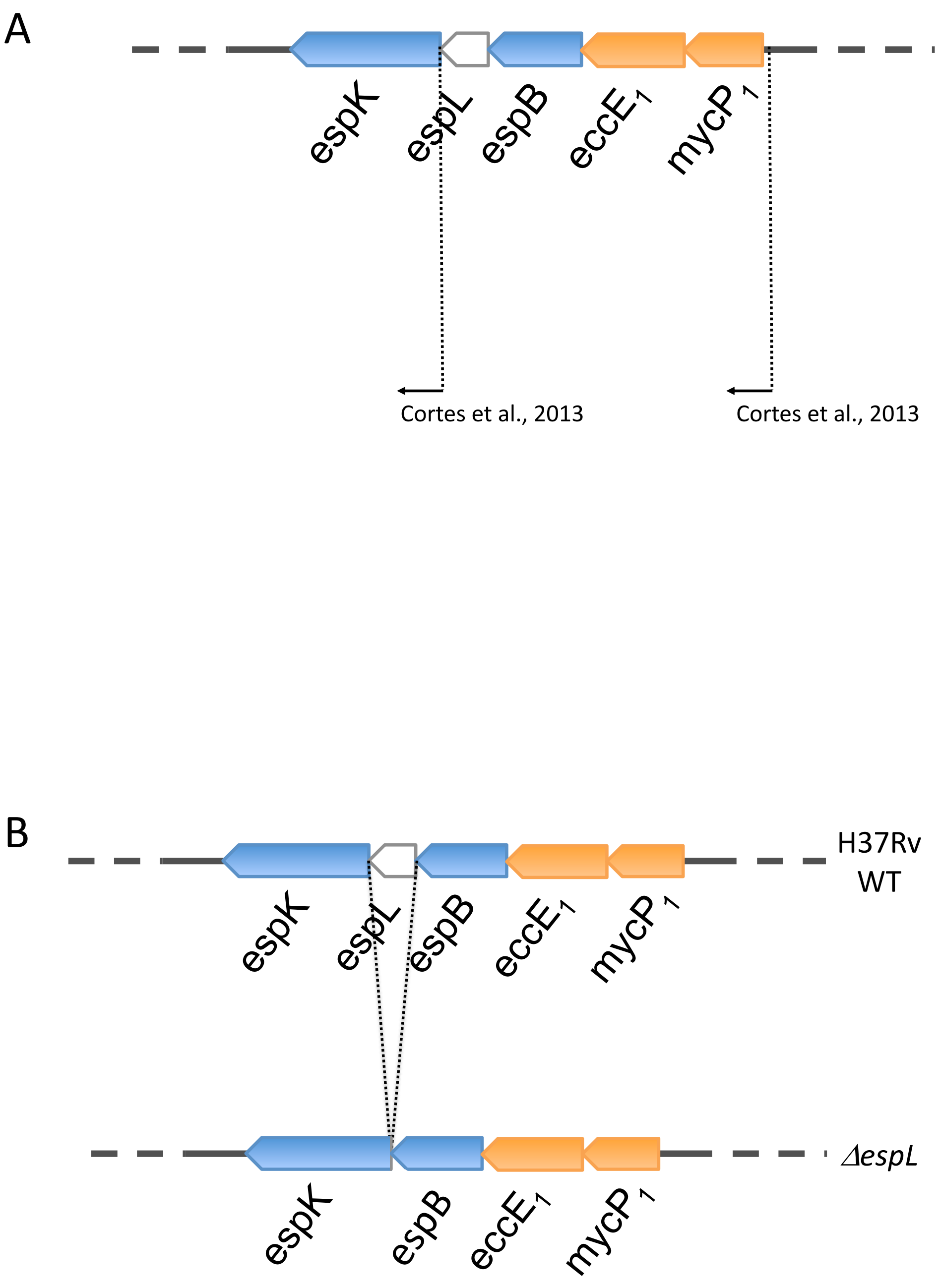

S1 Fig. Construction of Δ*espL* mutant. A) Schematic representation of the H37Rv genomic region that encompasses the *espL* gene. The 5'-ends of the mRNAs detected by Cortes and colleagues [1] are indicated by bent arrows. **B)** Construction of the Δ*espL* mutant by allelic exchange. An unmarked deletion was introduced into the *espL* native locus.

Bibliography

1. Cortes T, Schubert OT, Rose G, Arnvig KB, Comas I, Aebersold R, et al. Genome-wide Mapping of Transcriptional Start Sites Defines an Extensive Leaderless Transcriptome in Mycobacterium tuberculosis. Cell Rep. 2013;5: 1121–1131. doi:10.1016/j.celrep.2013.10.031
2. Cole ST, Brosch R, Parkhill J, Garnier T, Churcher C, Harris D, et al. Deciphering the biology of Mycobacterium tuberculosis from the complete genome sequence. Nature. 1998;393: 537–544. doi:10.1038/31159
3. Bottai D, Majlessi L, Simeone R, Frigui W, Laurent C, Lenormand P, et al. ESAT-6 secretion-independent impact of ESX-1 genes espF and espG1 on virulence of Mycobacterium tuberculosis. J Infect Dis. 2011;203: 1155–1164. doi:10.1093/infdis/jiq089
4. Chen JM, Boy-Röttger S, Dhar N, Sweeney N, Buxton RS, Pojer F, et al. EspD is critical for the virulence-mediating ESX-1 secretion system in Mycobacterium tuberculosis. J Bacteriol. 2012;194: 884–893. doi:10.1128/JB.06417-11
5. Lou Y, Rybniker J, Sala C, Cole ST. EspC forms a filamentous structure in the cell envelope of *M ycobacterium tuberculosis* and impacts ESX-1 secretion: Filamentous structure formation by EspC. Mol Microbiol. 2017;103: 26–38. doi:10.1111/mmi.13575
6. Gomez JE, Bishai WR. whmD is an essential mycobacterial gene required for proper septation and cell division. Proc Natl Acad Sci U S A. 2000;97: 8554–8559. doi:10.1073/pnas.140225297
7. Kolly GS, Boldrin F, Sala C, Dhar N, Hartkoorn RC, Ventura M, et al. Assessing the essentiality of the decaprenyl-phospho- D -arabinofuranose pathway in *M ycobacterium tuberculosis* using conditional mutants: Druggability of the *M. tuberculosis* DPA pathway. Mol Microbiol. 2014;92: 194–211. doi:10.1111/mmi.12546
