# Supplementary material for: EspL is essential for virulence and stabilizes EspE, EspF and EspH levels in Mycobacterium tuberculosis
Source: PLoS Pathog. 2018 Dec 20;14(12):e1007491. doi: 10.1371/journal.ppat.1007491 (PMC6319747; doi:10.1371/journal.ppat.1007491)
Supplement: S2 Fig — A) Growth curves obtained by measuring the optical density at 600 nm of the different strains grown in 7H9 medium at 37°C with shaking. B) Uptake of various bacterial strains by THP-1 cells. THP-1 cells were infected at multiplicity of infection (MOI) of 1. The number of intracellular bacteria was evaluated by CFU 3 h post-infection. Data were expressed as the mean and SD of two independent replicates. ΔΔRD1 carries a deletion of the extended ESX-1 locus. ns, not significant in one-way ANOVA followed by Tukey’s multiple comparison test. (PDF) [file ppat.1007491.s010.pdf]

A

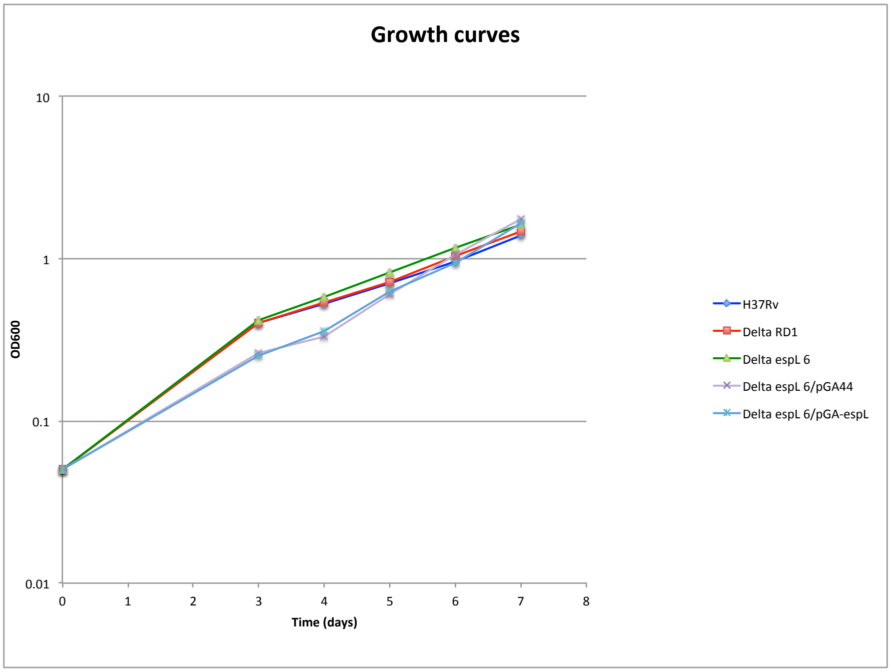

B

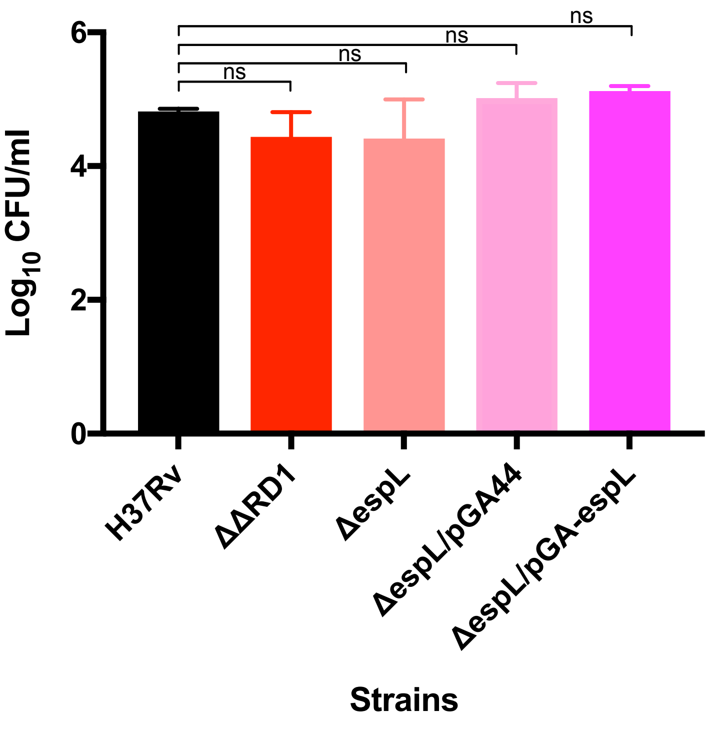

S2 Fig. Phenotypic analysis of Δ*espL* mutant. A) Growth curves obtained by measuring the optical density at 600 nm of the different strains grown in 7H9 medium at 37°C with shaking. **B)** Uptake of various bacterial strains by THP-1 cells. THP-1 cells were infected at multiplicity of infection (MOI) of 1. The number of intracellular bacteria was evaluated by CFU 3 h post-infection. Data were expressed as the mean and SD of two independent replicates. ΔΔRD1 carries a deletion of the extended ESX-1 locus. ns, not significant in one-way ANOVA followed by Tukey's multiple comparison test.
