# Supplementary material for: EspL is essential for virulence and stabilizes EspE, EspF and EspH levels in Mycobacterium tuberculosis
Source: PLoS Pathog. 2018 Dec 20;14(12):e1007491. doi: 10.1371/journal.ppat.1007491 (PMC6319747; doi:10.1371/journal.ppat.1007491)
Supplement: S3 Fig — A) Total cell lysates prepared from the indicated bacterial strains were analyzed by immunoblot. Membranes were probed for EsxA, EspB and GroEL2. B) Culture filtrates were analyzed as described for the total cell lysates. Antigen 85 (Ag85) represents the loading control. The experiment was repeated two times. One representative image is shown. (PDF) [file ppat.1007491.s011.pdf]

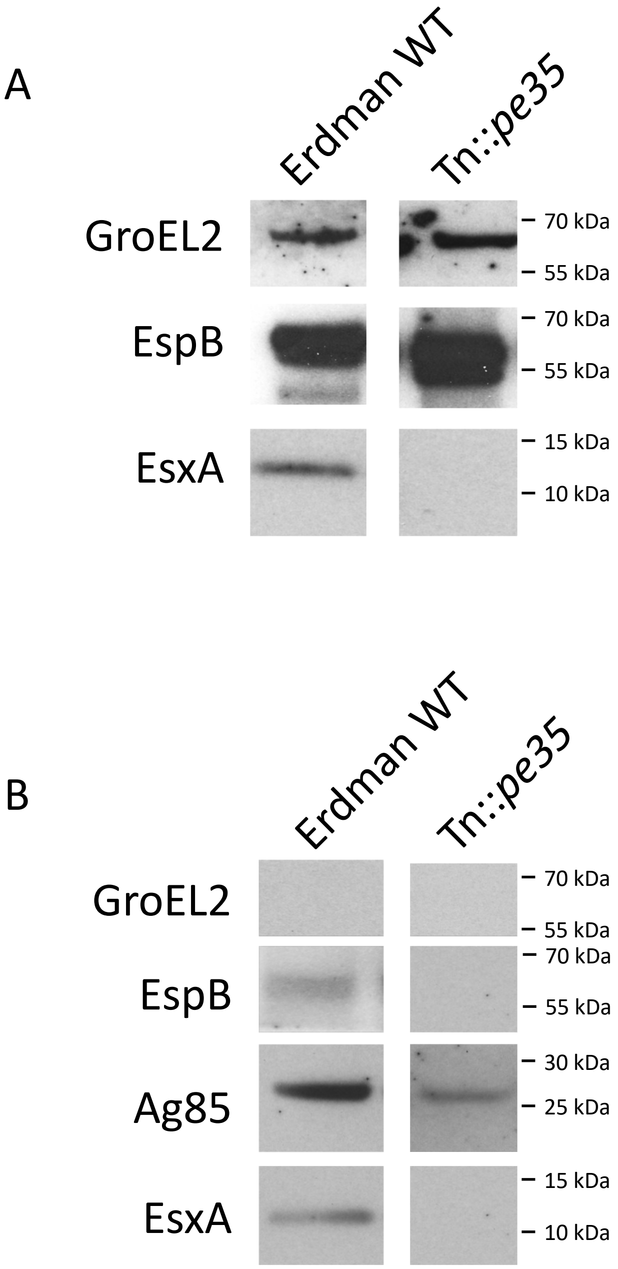

S3 Fig. Immunoblot analysis of EspB secretion. A) Total cell lysates prepared from the indicated bacterial strains were analyzed by immunoblot. Membranes were probed for EsxA, EspB and GroEL2. **B)** Culture filtrates were analyzed as described for the total cell lysates. Antigen 85 (Ag85) represents the loading control. The experiment was repeated two times. One representative image is shown.
