# Supplementary material for: EspL is essential for virulence and stabilizes EspE, EspF and EspH levels in Mycobacterium tuberculosis
Source: PLoS Pathog. 2018 Dec 20;14(12):e1007491. doi: 10.1371/journal.ppat.1007491 (PMC6319747; doi:10.1371/journal.ppat.1007491)
Supplement: S4 Fig — Volcano plot representation of the secretome comparison between ΔespL mutant and wild type strain. Blue lines indicate an FDR of 0.05 with a S0 = 0.5. Red points represent ESX-1 substrates. (PDF) [file ppat.1007491.s012.pdf]

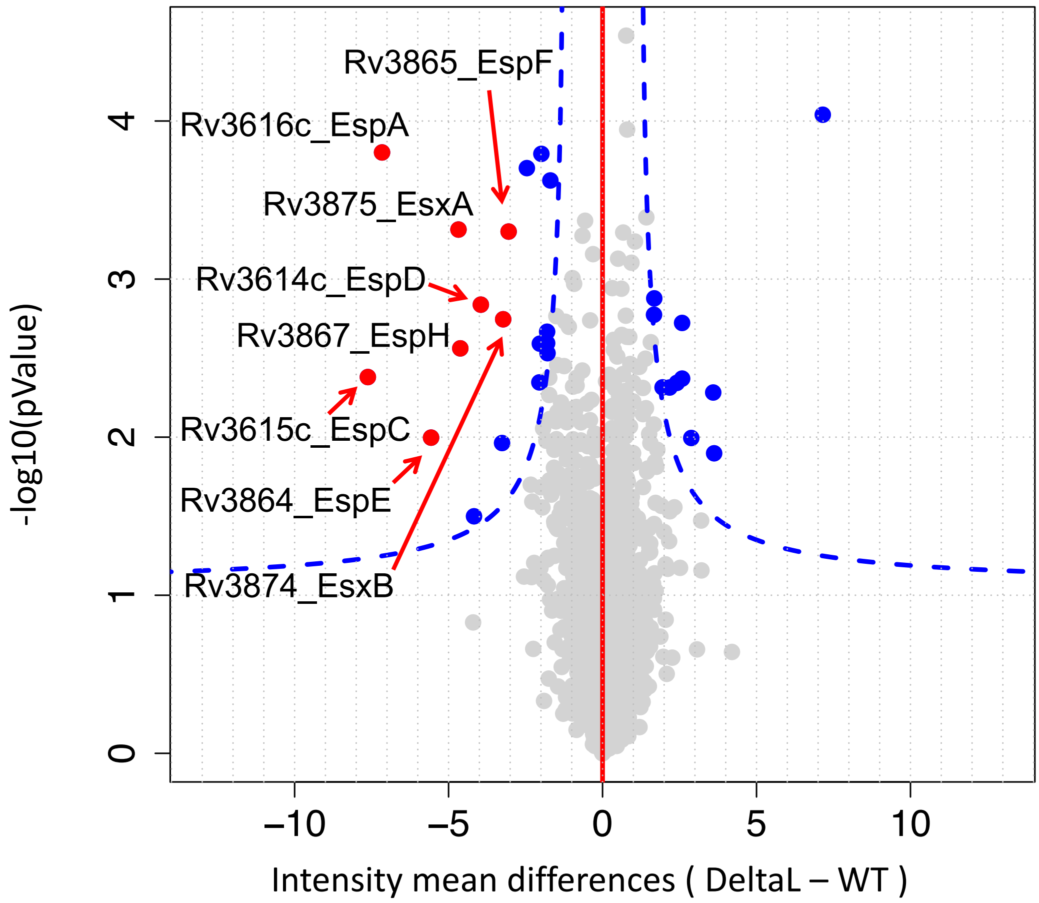

S4 Fig. Mass spectrometry analysis of the secretome of the Δ*espL* mutant. Volcano plot representation of the secretome comparison between Δ*espL* mutant and wild type strain. Blue lines indicate an FDR of 0.05 with a S0 = 0.5. Red points represent ESX-1 substrates which were found to be underrepresented in Δ*espL*.
