# Supplementary material for: EspL is essential for virulence and stabilizes EspE, EspF and EspH levels in Mycobacterium tuberculosis
Source: PLoS Pathog. 2018 Dec 20;14(12):e1007491. doi: 10.1371/journal.ppat.1007491 (PMC6319747; doi:10.1371/journal.ppat.1007491)
Supplement: S5 Fig — The culture filtrate, capsular, membrane and cytosolic fractions were analyzed by immunoblot. Membranes were probed for RpoB (cytosolic control), Rv3852 (membrane control), EsxB (culture filtrate control) and EspL. (PDF) [file ppat.1007491.s013.pdf]

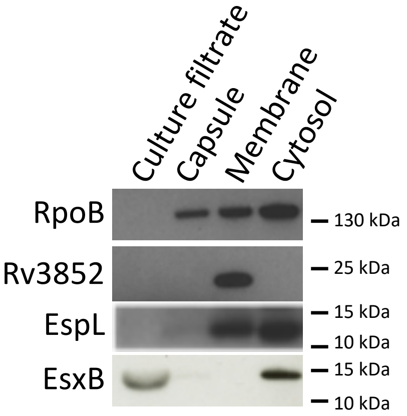

S5 Fig. Localization of EspL in subcellular fractions. The culture filtrate, capsular, membrane and cytosolic fractions were analyzed by immunoblot. Membranes were probed for RpoB (cytosolic control), Rv3852 (membrane control), EsxB (culture filtrate control) and EspL.
