# Supplementary material for: EspL is essential for virulence and stabilizes EspE, EspF and EspH levels in Mycobacterium tuberculosis
Source: PLoS Pathog. 2018 Dec 20;14(12):e1007491. doi: 10.1371/journal.ppat.1007491 (PMC6319747; doi:10.1371/journal.ppat.1007491)
Supplement: S6 Fig — qRT-PCR analysis was performed on total RNA extracted from the indicated strains. Expression levels of the various genes were obtained from two independent replicates, normalized to the housekeeping gene sigA and expressed as relative to H37Rv. **, p < 0.005. ns, not significant in two-way ANOVA followed by Tukey’s multiple comparison test. (PDF) [file ppat.1007491.s014.pdf]

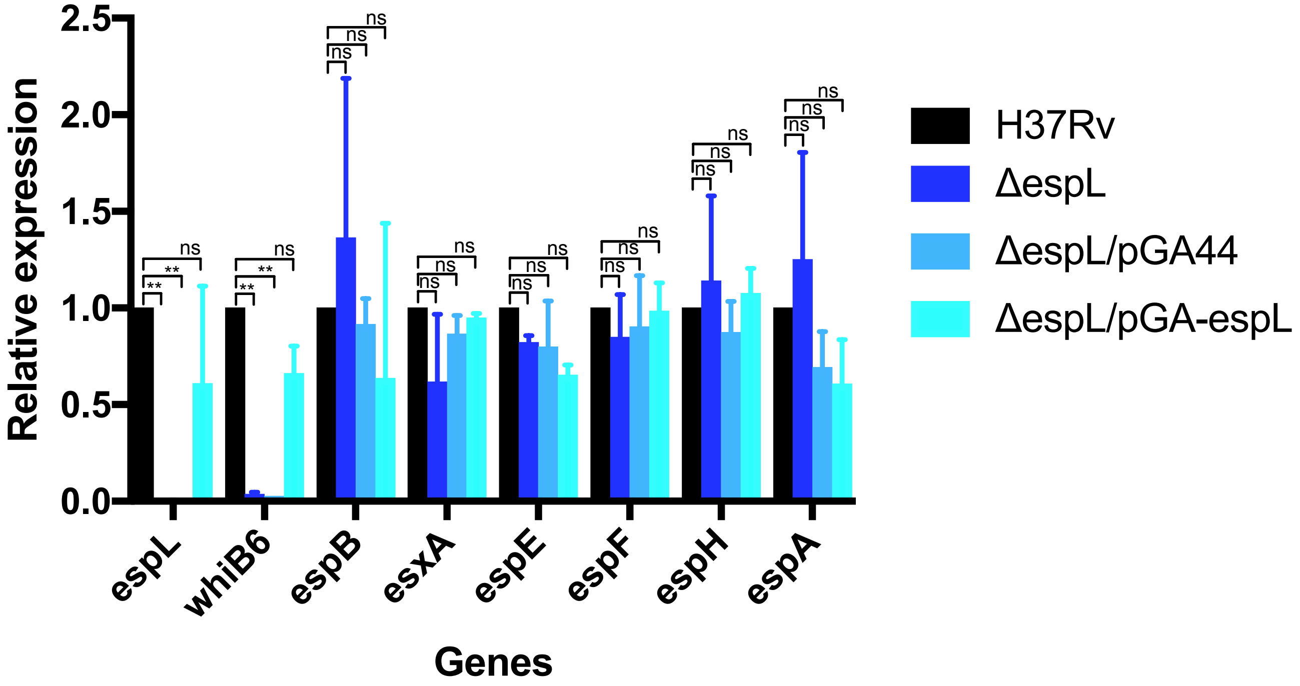

S6 Fig. Validation of RNA-seq results by qRT-PCR. qRT-PCR analysis was performed on total RNA extracted from the indicated strains. Expression levels of the various genes were obtained from two independent replicates, normalized to the housekeeping gene *sigA* and expressed as relative to H37Rv. **, $p < 0.005$. ns, not significant in two-way ANOVA followed by Tukey's multiple comparison test.
