# Supplementary material for: EspL is essential for virulence and stabilizes EspE, EspF and EspH levels in Mycobacterium tuberculosis
Source: PLoS Pathog. 2018 Dec 20;14(12):e1007491. doi: 10.1371/journal.ppat.1007491 (PMC6319747; doi:10.1371/journal.ppat.1007491)
Supplement: S7 Fig — Volcano plot representation of the total proteome comparison between ΔespL mutant and wild type strain. Blue lines indicate an FDR of 0.05 with a S0 = 0.1. Red points represent ESX-1 substrates that were found to be underrepresented in ΔespL. Green points represent EsxA and EsxB, which were more abundant in ΔespL compared to the wild type strain. (PDF) [file ppat.1007491.s015.pdf]

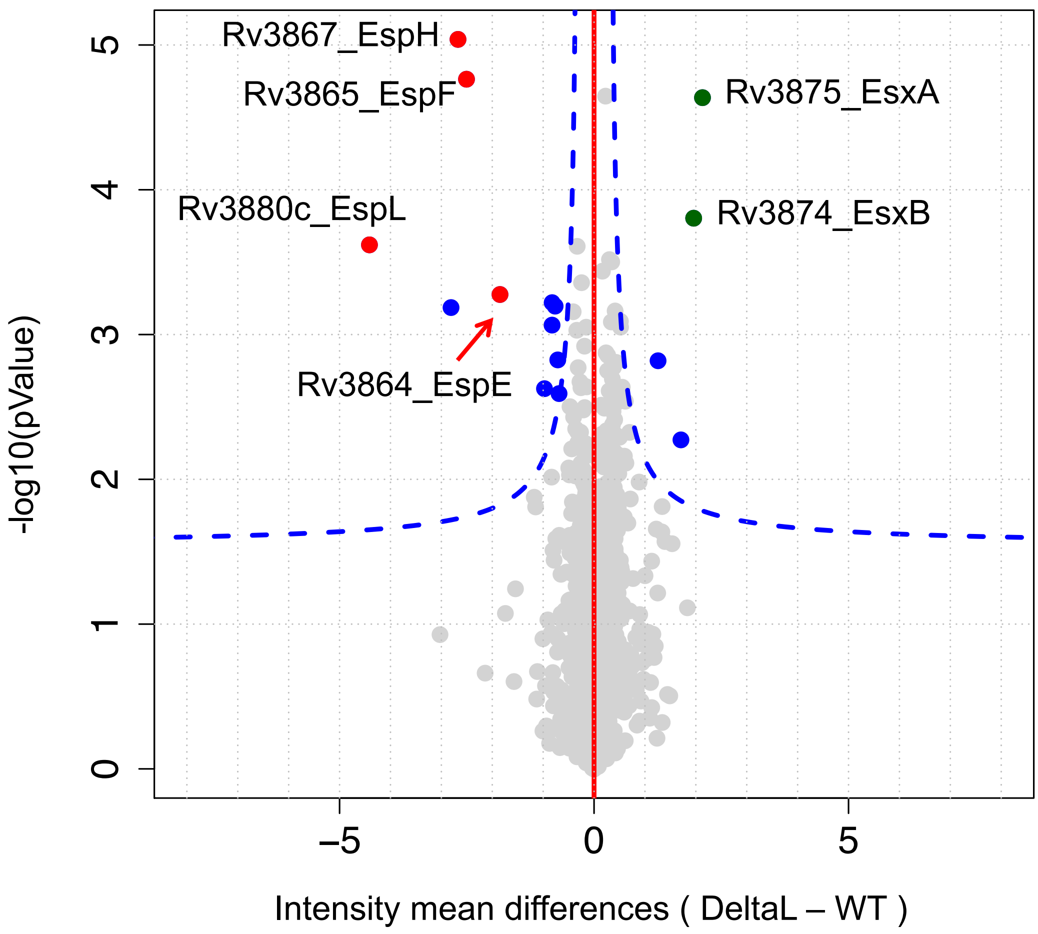

S7 Fig. Mass spectrometry analysis of the total proteome of the Δ*espL* mutant. Volcano plot representation of the total proteome comparison between Δ*espL* mutant and wild type strain. Blue lines indicate an FDR of 0.05 with a S0 = 0.1. Red points represent ESX-1 substrates that were found to be underrepresented in Δ*espL*. Green points represent EsxA and EsxB, which were more abundant in Δ*espL* compared to the wild type strain.
