# Supplementary material for: EspL is essential for virulence and stabilizes EspE, EspF and EspH levels in Mycobacterium tuberculosis
Source: PLoS Pathog. 2018 Dec 20;14(12):e1007491. doi: 10.1371/journal.ppat.1007491 (PMC6319747; doi:10.1371/journal.ppat.1007491)
Supplement: S8 Fig — A) qRT-PCR analysis of espL, whiB6, esxA, espE and espE.HA gene expression levels in different strains. Data were obtained from two independent replicates, normalized to the housekeeping gene sigA and expressed as relative to H37Rv/pGA-espE.HA. *, p < 0.05. **, p < 0.005. ns, not significant in two-way ANOVA followed by Tukey’s multiple comparison test. B) Virulence of ΔespL mutant expressing whiB6 in trans compared to H37Rv, ΔespL and complemented strain in the THP-1 infection model. THP-1 cells were infected at multiplicity of infection (MOI) of 5. Note that all of the strains, except ΔΔRD1, express espE.HA. ΔΔRD1 carries a deletion of the extended ESX-1 locus. Fluorescence measurements directly correlate with THP-1 viability. Data were expressed as the mean and standard deviation (SD) of four independent replicates. NI: not infected control. ****, p < 0.0001. ns, not significant in one-way ANOVA followed by Tukey’s multiple comparison test. C) qRT-PCR analysis of the expression levels of the indicated genes in different strains, upon ectopic expression of whiB6. Data were obtained from two independent replicates, normalized to the housekeeping gene sigA and expressed as relative to H37Rv/pGA-espE.HA. ****, p < 0.0001. ns, not significant in two-way ANOVA followed by Tukey’s multiple comparison test. (PDF) [file ppat.1007491.s016.pdf]

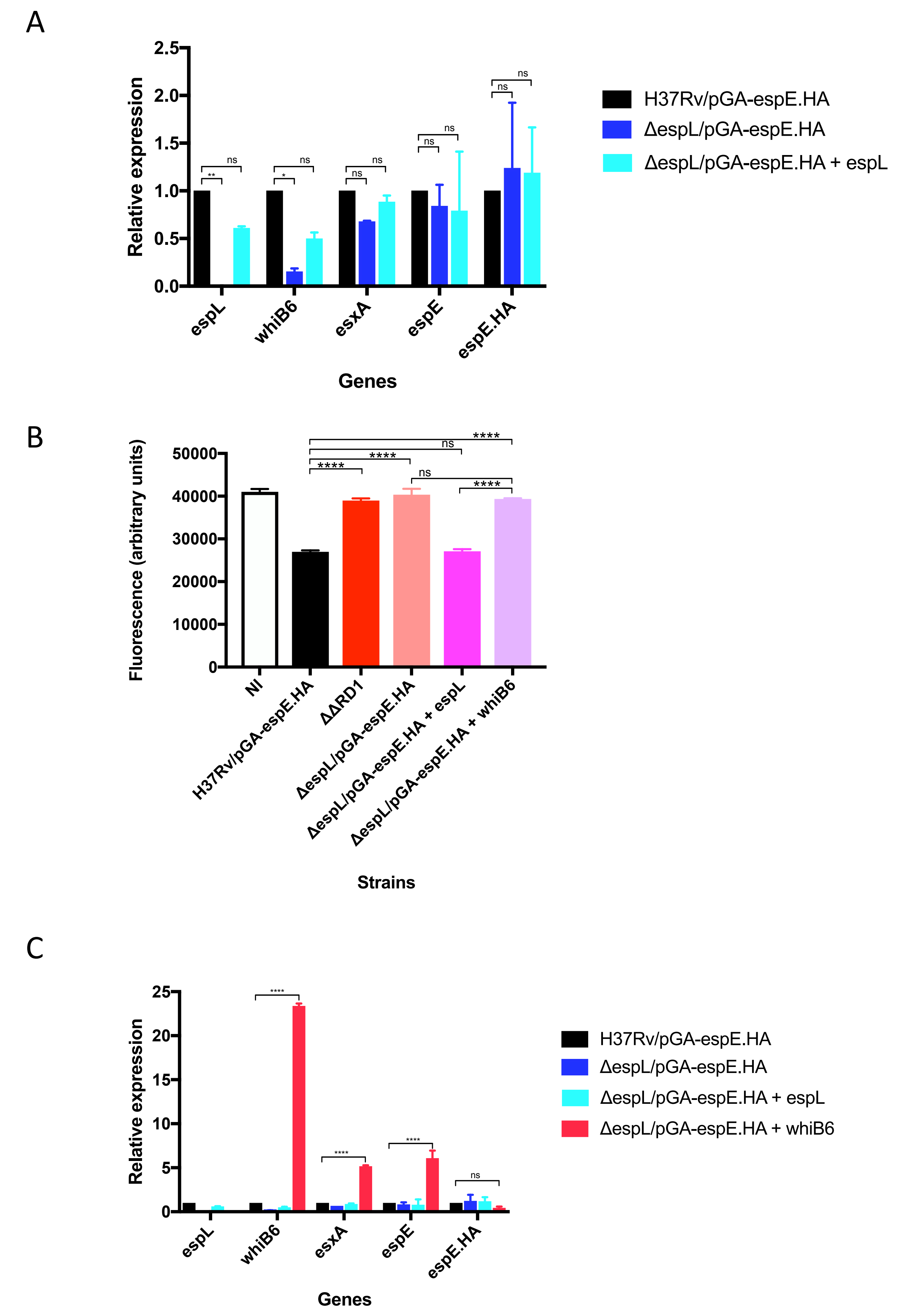

S8 Fig. Legend on next page.

S8 Fig. Expression of EspE.HA in H37Rv and in Δ*espL* mutant. A) qRT-PCR analysis of *espL*, *whiB6*, *esxA, espE* and *espE*.HA gene expression levels in different strains. Data were obtained from two independent replicates, normalized to the housekeeping gene *sigA* and expressed as relative to H37Rv/*pGA-espE*.HA. *, $p < 0.05$. **, $p < 0.005$. ns, not significant in two-way ANOVA followed by Tukey's multiple comparison test. **B)** Virulence of Δ*espL* mutant expressing *whiB6 in trans* compared to H37Rv, Δ*espL* and complemented strain in the THP-1 infection model. THP-1 cells were infected at multiplicity of infection (MOI) of 5. Note that all of the strains, except ΔΔRD1, express *espE*.HA. ΔΔRD1 carries a deletion of the extended ESX-1 locus. Fluorescence measurements directly correlate with THP-1 viability. Data were expressed as the mean and standard deviation (SD) of four independent replicates. NI: not infected control. ****, $p < 0.0001$. ns, not significant in one-way ANOVA followed by Tukey's multiple comparison test. **C)** qRT-PCR analysis of the expression levels of the indicated genes in different strains, upon ectopic expression of *whiB6*. Data were obtained from two independent replicates, normalized to the housekeeping gene *sigA* and expressed as relative to H37Rv/*pGA-espE*.HA. ****, $p < 0.0001$. ns, not significant in two-way ANOVA followed by Tukey's multiple comparison test.
