# Supplementary material for: EspL is essential for virulence and stabilizes EspE, EspF and EspH levels in Mycobacterium tuberculosis
Source: PLoS Pathog. 2018 Dec 20;14(12):e1007491. doi: 10.1371/journal.ppat.1007491 (PMC6319747; doi:10.1371/journal.ppat.1007491)
Supplement: S9 Fig — A) Immunoblot showing expression of EspL.HA and HA.EspL in total protein extracts from two independent clones (1 and 2) obtained upon transformation of ΔespL with a plasmid encoding espL.HA or HA.espL, respectively. Protein extract from espC::Tn/pMDespACHAD [24] was used as a control. B) Virulence of ΔespL mutant complemented by espL.HA or by HA.espL compared to H37Rv, ΔespL and complemented strain in the THP-1 infection model. ΔΔRD1 carries a deletion of the extended ESX-1 locus. THP-1 cells were infected at multiplicity of infection (MOI) of 5. Fluorescence measurements directly correlate with THP-1 viability. Data were expressed as the mean and standard deviation (SD) of four independent replicates. NI: not infected control. ****, p < 0.0001. ns, not significant in one-way ANOVA followed by Tukey’s multiple comparison test. (PDF) [file ppat.1007491.s017.pdf]

A

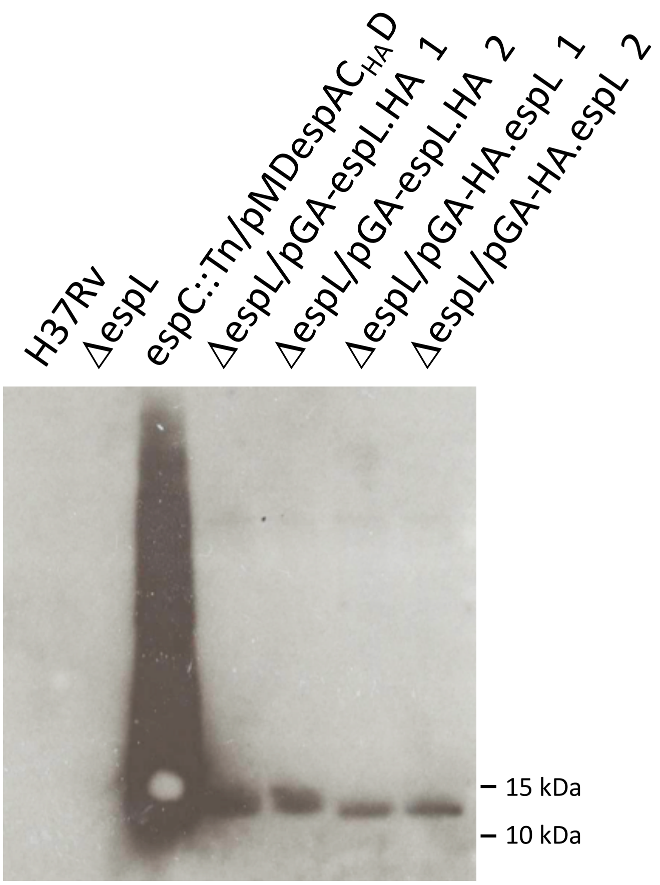

B

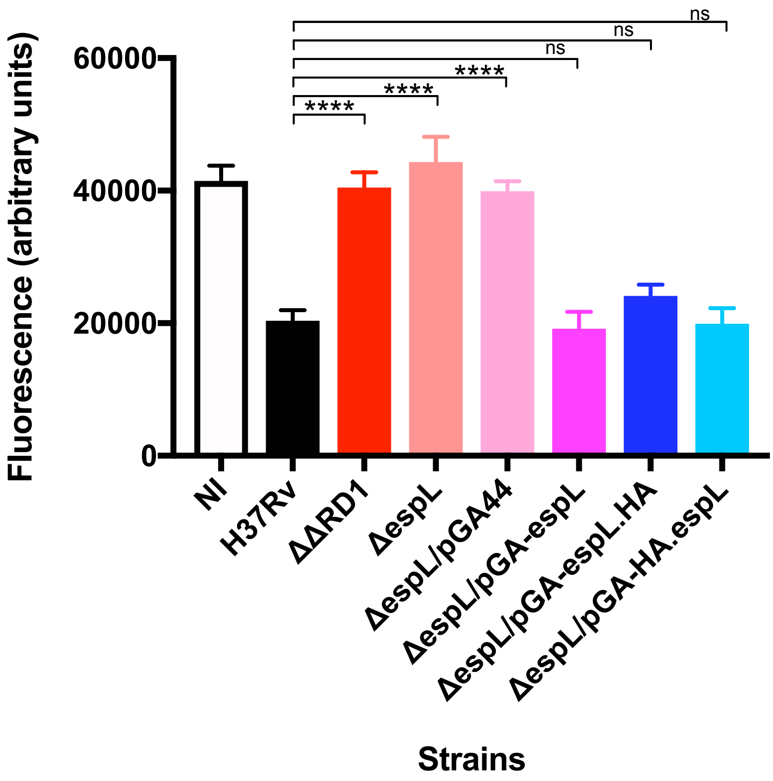

S9 Fig. Legend on next page.

S9 Fig. Validation of the expression of HA-tagged EspL and virulence analysis. A) Immunoblot showing expression of EspL.HA and HA.EspL in total protein extracts from two independent clones (1 and 2) obtained upon transformation of Δ*espL* with a plasmid encoding *espL*.HA or HA.*espL*, respectively. Protein extract from *espC::Tn*/pMD$espAC_{HA}D$ [4] was used as a control. **B)** Virulence of Δ*espL* mutant complemented by *espL*.HA or by HA.*espL* compared to H37Rv, Δ*espL* and complemented strain in the THP-1 infection model. ΔΔRD1 carries a deletion of the extended ESX-1 locus. THP-1 cells were infected at multiplicity of infection (MOI) of 5. Fluorescence measurements directly correlate with THP-1 viability. Data were expressed as the mean and standard deviation (SD) of four independent replicates. NI: not infected control. ****, $p < 0.0001$. ns, not significant in one-way ANOVA followed by Tukey's multiple comparison test.
